# Environmental Metagenomic Assemblies Reveal Seven New Highly Divergent Chlamydial Lineages and Hallmarks of a Conserved Intracellular Lifestyle

**DOI:** 10.3389/fmicb.2018.00079

**Published:** 2018-02-20

**Authors:** Trestan Pillonel, Claire Bertelli, Gilbert Greub

**Affiliations:** Center for Research on Intracellular Bacteria, Institute of Microbiology, Centre Hospitalier Universitaire Vaudois, Lausanne, Switzerland

**Keywords:** *Chlamydiae*, comparative genomics, bacterial taxonomy, chlamydial metabolism, endosymbiont evolution, obligate intracellular

## Abstract

The *Chlamydiae* phylum exclusively encompasses bacteria sharing a similar obligate intracellular life cycle. Existing 16S rDNA data support a high diversity within the phylum, however genomic data remain scarce owing to the difficulty in isolating strains using culture systems with eukaryotic cells. Yet, *Chlamydiae* genome data extracted from large scale metagenomic studies might help fill this gap. This work compares 33 cultured and 27 environmental, uncultured chlamydial genomes, in order to clarify the phylogenetic relatedness of the new chlamydial clades and to investigate the genetic diversity of the *Chlamydiae* phylum. The analysis of published chlamydial genomes from metagenomics bins and single cell sequencing allowed the identification of seven new deeply branching chlamydial clades sharing genetic hallmarks of parasitic *Chlamydiae*. Comparative genomics suggests important biological differences between those clades, including loss of many proteins involved in cell division in the genus *Similichlamydia*, and loss of respiratory chain and tricarboxylic acid cycle in several species. Comparative analyses of chlamydial genomes with two proteobacterial orders, the *Rhizobiales* and the *Rickettsiales* showed that genomes of different *Rhizobiales* families are much more similar than genomes of different *Rickettsiales* families. On the other hand, the chlamydial 16S rRNAs exhibit a higher sequence conservation than their *Rickettsiales* counterparts, while chlamydial proteins exhibit increased sequence divergence. Studying the diversity and genome plasticity of the entire *Chlamydiae* phylum is of major interest to better understand the emergence and evolution of this ubiquitous and ancient clade of obligate intracellular bacteria.

## Introduction

Bacteria of the phylum *Chlamydiae* are all obligate intracellular bacteria that multiply within eukaryotic host cells (Horn, 2015). Various chlamydial species were identified in mammals, birds, reptiles, fishes, arthropods, and unicellular eukaryotes (Taylor-Brown et al., 2015). Several *Chlamydiae*, such as *Chlamydia trachomatis, Chlamydia abortus*, and *Chlamydia pneumoniae*, are well-known important human and animal pathogens (Elwell et al., 2016). Other *Chlamydia*-related organisms such as *Waddlia chondrophila*, associated with abortion in cattle, are increasingly recognized as emerging pathogens posing a zoonotic or vector-borne risk (Taylor-Brown and Polkinghorne, 2017). Multiple species of the *Simkaniaceae*, “*Candidatus* Piscichlamydiaceae” and “*Ca*. Clavichlamydiaceae” are associated with epitheliocystis, a disease affecting the gill of fish. *Rhabdochlamydiaceae* spp. are also highly prevalent in ticks, the most common arthropod vector of human and animal disease (Pilloux et al., 2015). Analysis of environmental 16S rRNA sequences indicates that those organisms are highly diverse and are present in a wide variety of freshwater, marine and terrestrial environments (Lagkouvardos et al., 2014). However, there are currently only six validated families (*Chlamydiaceae, Parachlamydiaceae, Simkaniaceae, Waddliaceae, Criblamydiaceae*, and *Rhabdochlamydiaceae*) and three *Candidatus* families (Clavichlamydiaceae, Parilichlamydiaceae, and Piscichlamydiaceae) (Stride et al., 2013; Horn, 2015).

The last comparative analysis of the phylum *Chlamydiae* (Collingro et al., 2011) included genomic data from four families; a single species of *Waddliaceae* and *Simkaniaceae*, and multiple species of *Chlamydiaceae* and *Parachlamydiaceae*. This work reported a relatively large set of 560 genes conserved in all four chlamydial clades, and a large number of accessory genes restricted to families exhibiting much larger genomes than vertebrate parasites of the *Chlamydia* genus. Some virulence factors such as the Type III secretion system were shown to be conserved in all four families investigated. On the other hand, the F-like conjugative DNA transfer system identified by Greub et al. (2004) in *Protochlamydia amobophila* was restricted to *Simkaniaceae* and *Parachlamydiaceae* (Bertelli et al., 2016). Since then, multiple genomes belonging to the family *Parachlamydiaceae* were sequenced from strains in pure culture, including one representative of a new genus named *Rubidus* (Domman et al., 2014; Ishida et al., 2014; Yamaguchi et al., 2015; Yamane et al., 2015; Bertelli et al., 2016; Bou Khalil et al., 2016, 2017; Fukumoto et al., 2016). In addition, genome sequences representing two distinct genera of the family *Criblamydiaceae* were recently released (Bertelli et al., 2014, 2015).

Previously, technically challenging and time-consuming *in vitro* cultures were required to obtain sufficient sample material for genomic analysis of *Chlamydiae* (Jacquier et al., 2013). This difficulty was recently overcome by culture-independent genomic characterization of uncultivable *Chlamydiae*. In 2013, the genome of *C. trachomatis* could be recovered directly from clinical samples using a metagenomic approach (Andersson et al., 2013; Seth-Smith et al., 2013). More recently, the complete genome of “*Candidatus* Chlamydia sanzinia”—an uncultivated snake pathogen—was sequenced without culture (Taylor-Brown et al., 2016). The fish pathogen “*Candidatus* Similichlamydia epinepheli,” a representative of the *Candidatus* family Parilichlamydiaceae, was partially recovered using a similar approach (Taylor-Brown et al., 2017). Moreover, several “*Candidatus* Rhabdochlamydia spp.” genomes could also be directly sequenced from ticks samples (Pillonel et al., unpublished data). Single-cell genomics is another promising approach for the study of uncultivable organisms (Gawad et al., 2016). This technique allowed the recovery of partial genomes of three new chlamydial lineages from marine environments (Collingro et al., 2017).

Thus, from 2011 to 2017, a large number of additional chlamydial genomes have been made available but their diversity and their relative position in the phylogenetic tree of the phylum *Chlamydiae* remained so far unknown. Updated comparative genomic analyses will allow to clarify the phylogenetic relatedness of the new chlamydial clades and to improve our understanding of the evolution of shared and distinct genetic features in this diverse group of obligate intracellular bacteria.

Therefore, we performed here a comprehensive comparative analysis of the phylum *Chlamydiae* including all genomic data currently publicly available (as of June 2017). We have identified 7 putative novel family-level lineages retrieved from metagenomics bins. Extremely diverse genomic characteristics were observed, with genome size ranging from 1 to 3.4 Mbp and GC content ranging from 26.23 to 55.76%. Despite those variations, essential mechanisms involved in host-symbiont interactions such as the type III secretion apparatus and the ADP-ATP translocase (a transporter involved in energy parasitism) are conserved in all newly discovered lineages. On the other hand, fundamental biological differences such as distinct division mechanisms of novel and phylogenetically distantly-related *Chlamydiae* could be highlighted.

## Methods

### Data retrieval

Genome assemblies were downloaded from the RefSeq database, and from Genbank for the assemblies absent from RefSeq (Table 1; O'Leary et al., 2016). All genome assemblies from the phylum *Chlamydiae* (NCBI taxonomy ID 204428) were included in this study with the exception of the *Chlamydia* genus. Since several *Chlamydia* species are well-studied pathogens with many sequenced genomes, a single representative genome assembly was considered for each of the 13 *Chlamydia* species out of a total of 326 genome assemblies (June 2017).

**Table 1:** List of genomes included in the analysis, with GC content (%), size (bp) and completeness as evaluated using checkM.

**Accession**	**Description**	**Size (bp)**	**Number of CDS**	**GC (%)**	**Number of contigs**	**Completeness**
NC_010655	*Akkermansia muciniphila* ATCC BAA-835	2664102	2,246	55.76	1	100
NC_004552	*Chlamydia abortus* S26/3	1144377	935	39.87	1	98.28
NZ_CP006571	*Chlamydia avium* 10DC88	1041170	842	36.92	1	94.83
NC_003361	*Chlamydia caviae* GPIC	1173390	968	39.22	1	98.28
NC_007899	*Chlamydia felis* Fe/C-56 Fe/C-56	1166239	966	39.38	1	98.28
NZ_CP015840	*Chlamydia gallinacea* 08-1274/3	1059583	905	37.94	1	96.55
NZ_APJW00000000	*Chlamydia ibidis* 10-1398/6	1146066	939	38.32	4	96.55
NC_002620	*Chlamydia muridarum* Nigg	1072950	900	40.34	1	98.28
NC_015408	*Chlamydia pecorum* E58	1106197	934	41.08	1	100
NC_000922	*Chlamydia pneumoniae* CWL029	1230230	1,029	40.58	1	98.28
NC_015470	*Chlamydia psittaci* 6BC	1171660	983	39.06	1	98.28
NZ_CP014639	*Chlamydia sanzinia* 2742-308	1113233	933	38.54	1	98.28
MKSG01000000	*Chlamydia* sp. 32-24	2529957	2,075	32.42	98	96.55
NZ_AYKJ01000000	*Chlamydia suis* MD56	1073507	886	42.03	13	98.28
NC_000117	*Chlamydia trachomatis* D/UW-3/CX	1042519	887	41.31	1	98.28
LNES01000000	Chlamydiae bacterium Ga0074140	1724203	1,639	47.82	6	96.55
MGLO01000000	Chlamydiae bacterium GWA2_50_15	1177368	993	49.34	32	93.1
MGLP01000000	Chlamydiae bacterium GWC2_50_10	1172283	966	48.94	52	86.21
MGLQ01000000	Chlamydiae bacterium GWF2_49_8	1019733	760	49.23	93	77.9
MGLR01000000	Chlamydiae bacterium RIFCSPHIGHO2_01_FULL_44_39	1569649	1,466	44.72	30	95.69
MGLS01000000	Chlamydiae bacterium RIFCSPHIGHO2_02_FULL_45_9	1342635	1,156	44.66	104	80.72
MGLT01000000	Chlamydiae bacterium RIFCSPHIGHO2_02_FULL_49_29	1387015	1,187	49.08	69	93.1
MGLU01000000	Chlamydiae bacterium RIFCSPHIGHO2_12_FULL_27_8	974360	817	27.43	98	72.1
MGLV01000000	Chlamydiae bacterium RIFCSPHIGHO2_12_FULL_44_59	1568548	1,470	44.72	30	95.69
MGLW01000000	Chlamydiae bacterium RIFCSPHIGHO2_12_FULL_49_11	1258484	1,065	48.45	71	81.27
MGLX01000000	Chlamydiae bacterium RIFCSPHIGHO2_12_FULL_49_32	1397302	1,190	48.91	72	89.66
MGLY01000000	Chlamydiae bacterium RIFCSPHIGHO2_12_FULL_49_9	1320387	1,166	48.59	94	71.24
MGLZ01000000	Chlamydiae bacterium RIFCSPLOWO2_01_FULL_28_7	708526	572	28.07	75	58.21
MGMA01000000	Chlamydiae bacterium RIFCSPLOWO2_01_FULL_44_52	1537590	1,438	44.74	29	95.69
MGMB01000000	Chlamydiae bacterium RIFCSPLOWO2_02_FULL_45_22	1575869	1,475	44.7	30	95.69
MGMC01000000	Chlamydiae bacterium RIFCSPLOWO2_02_FULL_49_12	1413329	1,175	48.99	58	93.1
MGMD01000000	Chlamydiae bacterium RIFCSPLOWO2_12_FULL_45_20	1542182	1,443	44.75	28	95.69
MGME01000000	Chlamydiae bacterium RIFCSPLOWO2_12_FULL_49_12	1418742	1,224	49.16	46	91.38
LJUH01000000	Chlamydiae bacterium SM23_39	1126604	986	26.23	67	96.55
MKSK01000000	Chlamydiales bacterium 38-26	2834110	2,327	38.12	8	98.28
NZ_FLYF00000000	Chlamydiales bacterium SCGC AB-751-O23	986924	715	35.45	89	46.24
NZ_FLYO00000000	Chlamydiales bacterium SCGC AG-110-M15	929815	711	41.8	54	40.52
NZ_FLYP00000000	Chlamydiales bacterium SCGC AG-110-P3	1299661	954	46.83	96	47.49
NZ_CCEJ000000000	Criblamydia sequanensis CRIB-18	2969604	2,422	38.24	23	97.41
CWGJ01000001	*Estrella lausannensis* CRIB-30	2820195	2,202	48.23	29	97.41
NZ_JSDQ00000000	Neochlamydia sp. EPS4	2530677	1,882	38.09	112	97.41
NZ_BASK00000000	Neochlamydia sp. S13	3187074	2,232	38.03	1,342	97.41
NZ_JRXI00000000	Neochlamydia sp. TUME1	2546323	1,879	38.02	254	97.41
NZ_BAWW00000000	*Parachlamydia acanthamoebae* Bn9	2999361	2,409	38.94	72	99.66
NZ_ACZE00000000	*Parachlamydia acanthamoebae* Halls coccus	2971261	2,477	38.97	95	99.66
NZ_JSAM00000000	*Parachlamydia acanthamoebae* OEW1	3008885	2,309	39.04	162	97.07
NC_015702	*Parachlamydia acanthamoebae* UV-7	3072383	2,532	39.03	1	99.66
NZ_BBPT00000000	*Parachlamydiaceae bacterium* HS-T3	2307885	2,025	38.71	34	100
NZ_JSAN00000000	*Protochlamydia amoebophila* EI2	2397675	1,775	34.82	178	100
NC_005861	*Protochlamydia amoebophila* UWE25	2414465	1,841	34.72	1	98.28
NZ_CCJF00000000	*Protochlamydia naegleriophila* Diamant	2864073	2,359	42.81	4	98.28
NZ_LN879502	*Protochlamydia naegleriophila* KNic	2885090	2,372	42.7	1	100
NZ_FCNU00000000	*Protochlamydia phocaeensis*	3423982	2,766	42.05	33	100
NZ_BASL00000000	Protochlamydia sp. R18 S13	2722699	2,006	34.78	795	100
NZ_BCPZ00000000	Protochlamydia sp. W-9	2484573	1,815	34.48	402	100
PRJEB24578	*Rhabdochlamydia helvetica* T3358	1830543	1,692	36.16	38	100
CCSC01000000	*Rubidus massiliensis*	2701449	2,299	32.45	3	98.28
PRJNA343727	*Similichlamydia epinepheli* GCCT14	981540	913	39.52	169	70
NC_015713	*Simkania negevensis* Z	2496337	2,229	41.78	1	100
NC_014225	*Waddlia chondrophila* WSU 86-1044	2116312	1,832	43.78	1	98.28

### Evaluation of genome completeness and quality

Many genomes included in this work are metagenomics bins that may be of unequal quality. Indeed, metagenomics bins can be an incomplete representation of the organism's genomes, can be a mixture of multiple genomes or include chimeric DNA sequences. To screen for contaminants, the completeness and redundancy of each genome was thus evaluated based on the identification of 104 nearly universal (and generally single copy) bacterial markers with checkM (Parks et al., 2015). In addition, translated predicted coding sequences (CDS) of each genome were compared to RefSeq database using PLAST, a fast sequence similarity search tool, with the following parameters: -M BLOSUM62 -s 45 -seeds-use-ratio 60 -G 11 -E 1 -F F -max-hit-per-query 100 -max-hsp-per-hit 1 (Van Nguyen and Lavenier, 2009). PLAST was favored over BLAST for its lower memory usage. The taxonomy of the first PLAST hit (excluding hits against RefSeq sequences classified as belonging to the same species) was investigated with help of the NCBI taxonomy database (Federhen, 2012).

### Comparative genome analysis and reconstruction of the species phylogeny

Orthologs were identified using OrthoFinder version 0.4 (Emms and Kelly, 2015). A reference phylogeny was built based on single copy orthologs conserved in at least 55 of the 60 studied genomes (Table 1). Core single copy orthologs were aligned using mafft version 7.058b (Katoh and Standley, 2013). The concatenated alignment was used for the reconstruction of the species tree using FastTree 2.1.9 with double precision (Price et al., 2010). Circular plots were drawn using Circos version 0.69 (Krzywinski et al., 2009), whereas other plots were made with R (R Core Team, 2016).

### Genome annotation and identification of secretion systems, flagellar subunits, and selected metabolic traits

All genomes were annotated using GhostKOALA (Kanehisa et al., 2016) and Interproscan version 5.23–62.0 (Mitchell et al., 2015). Orthologs of the type III and type IV secretion systems, known effectors, division proteins, membrane proteins, the Euo master regulator, respiratory chain complexes and enzymes involved in menaquinone biosynthesis and glycogen metabolism were identified based on OrthoFinder grouping into groups of orthologous proteins (see Table S1 for the detailed reference locus list). The ATP:ADP antiporters homologs were identified by InterProScan annotation (interpro accession: IPR004667). The flagellar apparatus subunits, proteins involved in peptidoglycan, purine, pyrimidine and ubiquinone biosynthesis as well as the glycolysis pathway, the citrate cycle (TCA), and the pentose phosphate pathway (PPP) were identified based on KEGG ortholog (KO) annotation. Reported counts for each pathway/module are the non-redundant number of identified KO. Predicted coding sequences were compared to COG database (version 2014) (Galperin et al., 2015) using BLASTP version 2.3.0+(Camacho et al., 2009) with an e-value cutoff of 1e^−5^, a minimal query coverage of 50% and a minimal identity of 20%.

### Identification of phylogenetic markers and evaluation of genetic relatedness

Proteins conserved between pairs of genomes were identified by pairwise protein sequences comparisons with BLASTP version 2.3.0+ (Camacho et al., 2009). The identity of all reciprocal best blast hit (RBBH) with a minimal e-value of 10^−5^ and 50% of both query and hit coverage were retained for the comparative analyses. 16S and 23S rRNA sequences were identified using barrnap (https://github.com/tseemann/barrnap). HMM profiles were built from 21 reference sequences for nine previously identified phylogenetically informative markers (Pillonel et al., 2015). These profiles were used to identify markers in all chlamydial genomes included in this study using hmmsearch v3.1 (Eddy, 2011). For each marker, a phylogenetic tree was reconstructed based on the best hmmsearch hit in each genome. Bitscore cut-offs were defined individually for each marker based on the density distribution of hmmsearch bit scores and gene tree topologies: DnaA (260), SucA (750), the hypothetical protein 325 (350), FabI (400), RpoN (300), FtsK (750), PepF (470), Adk (175), and HemL (360). Pairwise amino acid sequence identities were calculated based on pairwise Needleman-Wunsch global alignments made with Needle from the EMBOSS package version 6.6 (Rice et al., 2000). Gaps were not considered in identity calculations. The probability distributions of pairwise amino acid identity values were estimated using the statistical package R with kernel density estimations (R Core Team, 2016). Density plots were made using the ggplot2 package (Wickham, 2016).

## Results

### Twelve nearly complete genome assemblies from multiple new deeply branching *Chlamydiae* clades

In total, 60 genomes (including one outgroup) were compared in this study: 21 poorly characterized metagenomics bins classified as *Chlamydiae* were retrieved from Genbank. Most (*n* = 17) originate from a single study of groundwater microbial ecosystems (Anantharaman et al., 2016). Others were sequenced as part of the microbial community of a biologically active filter of a water treatment plant (*n* = 1) (Pinto et al., 2016), as part of an investigation of bacterial communities in estuary sediments (*n* = 1) (Baker et al., 2015), and from an experimental bioreactor used to treat contaminated goldmine water (*n* = 2) (Kantor et al., 2015). In addition, three single-cell amplified partial genomes from marine *Chlamydiae* (Collingro et al., 2017) and 25 previously published chlamydial genomes (Table 1) were studied. The latter contained a majority (*n* = 20) of draft assemblies. The verrucomicrobial genome of *Akkermansia muciniphila* was included as an outgroup.

**Figure 1:**
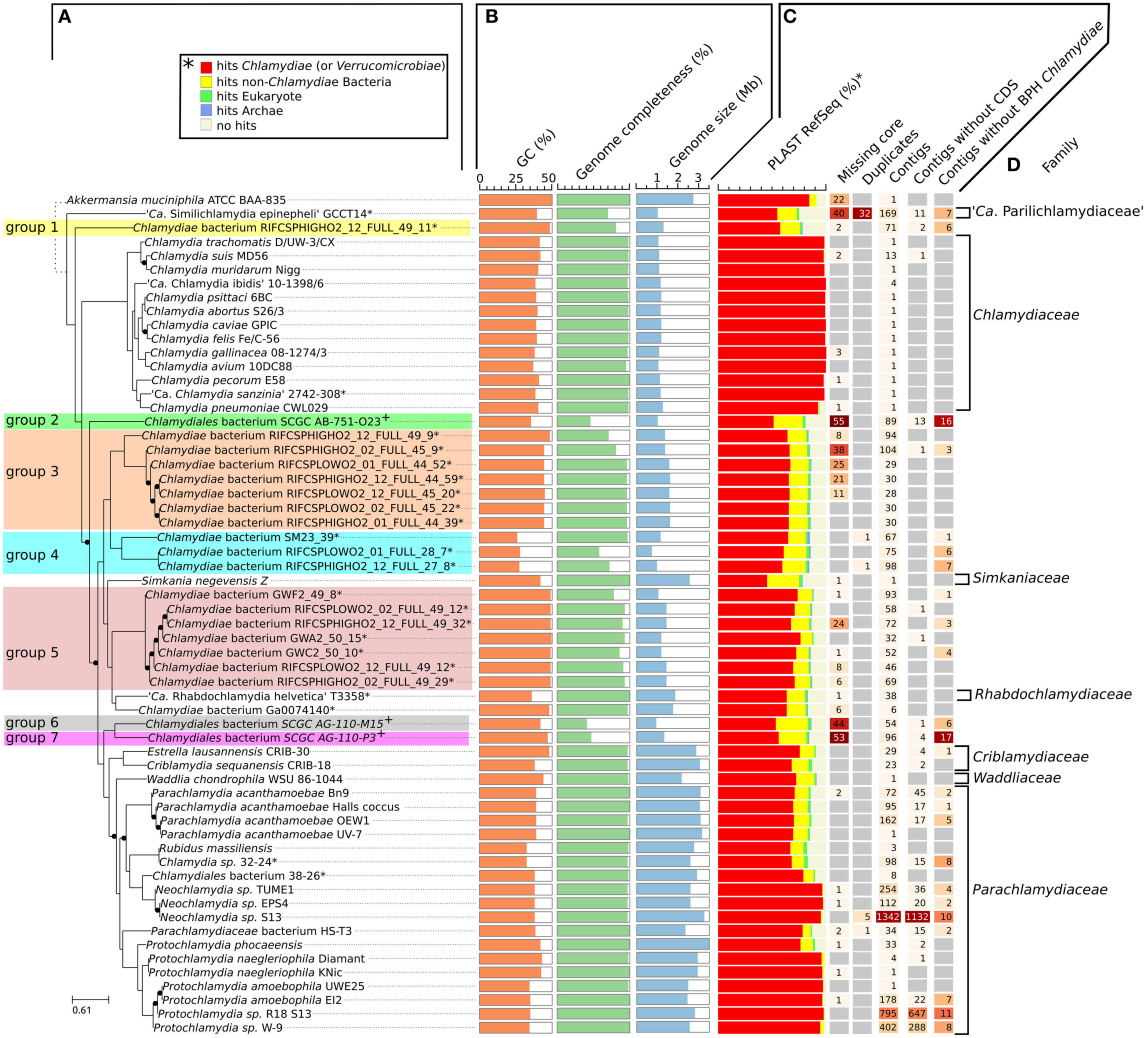
Diversity of the phylum *Chlamydiae*. **(A)** Phylogenetic tree of cultured and uncultured representative of the phylum *Chlamydiae* reconstructed based on the concatenated alignment of 108 single copy orthologs conserved in more than 90% of the genomes. Nodes with support values lower than 1 are indicated with black dots. The width of the black dots does not reflect a genetic distance (lines a shifted to accommodate the presence of the dots). *Genome sequenced using a shotgun metagenomics approach. ^+^Single-cell amplified genomes **(B)** GC content, genome size and estimated genome completeness **(C)** quality measures: (i) number of missing core proteins (out of 108 proteins) (ii) number of duplicated core genes (iii) number of contigs in the assembly (iv) number of contigs without any CDS (v) number of contigs without any best PLAST hits against chlamydial sequences (RefSeq database version 81). **(D)** Described families of the *Chlamydiae* phylum.

While most *Chlamydiae* share a similar life cycle, their genetic diversity is considerable. Standard procedures for the classification of *Chlamydiae* rely on a small set of reference genes or protein sequences, including the 16S rRNA sequence (Pillonel et al., 2015; Greub, 2017). Given that metagenomic datasets frequently lack rRNA operons, pairwise comparisons of all protein sequences were undertaken to better characterize the genetic diversity of newly sequenced genomes. The protein sequences of the 59 chlamydial genomes included in this comparative analysis were clustered into 10,162 orthologous groups (Table S2, Figure S1A). Among those groups, 4,553 are restricted to a single genome (44.8%, Figure S1B). Only three single copy orthologs were conserved in all chlamydial genomes (the two ribosomal proteins S8 and L6, and the tRNA threonylcarbamoyladenosine biosynthesis protein TsaE), but 108 orthologous groups were conserved in more than 90% of the genomes (55 out of 60, including the outgroup genome.The list of 108 orthologous groups is reported in Table S3). The concatenated alignment of those 108 protein sequences was used to build a reference phylogeny of all representative genomes of the phylum *Chlamydiae* (Figure 1A).

The GC content of uncultured strains varies widely from 26.2 to 49.3% (Table 1, Figure 1B). Twelve of the metagenomics bins are predicted to be more than ninety percent complete, based on the identification of 104 nearly universal bacterial proteins (Table 1, Table S4, Figure 1B). Those nearly complete assemblies range from 1.42 to 2.83 Mb in size. The largest metagenomics bin (*Chlamydia* sp. 32–24), is phylogenetically related to *Neochlamydia* spp. The smallest one, *Chlamydiae* bacterium RIFCSPLOWO2_01_FULL_28_7, only comprises about 700 kb that were predicted to represent approximately 58% of the genome. The most incomplete genomes are the three genomes from marine *Chlamydiae* (Figure 1, groups 2, 6, and 7). Those genomes are amplified from a single cell. Such approach frequently yield incomplete genomes (Collingro et al., 2017). *Chlamydiales* bacterium SCGC AG-110-M15 missed 55 of the 108 core proteins used to build the species phylogeny (50.9% of the dataset, Figure 1C).

**Figure 2:**
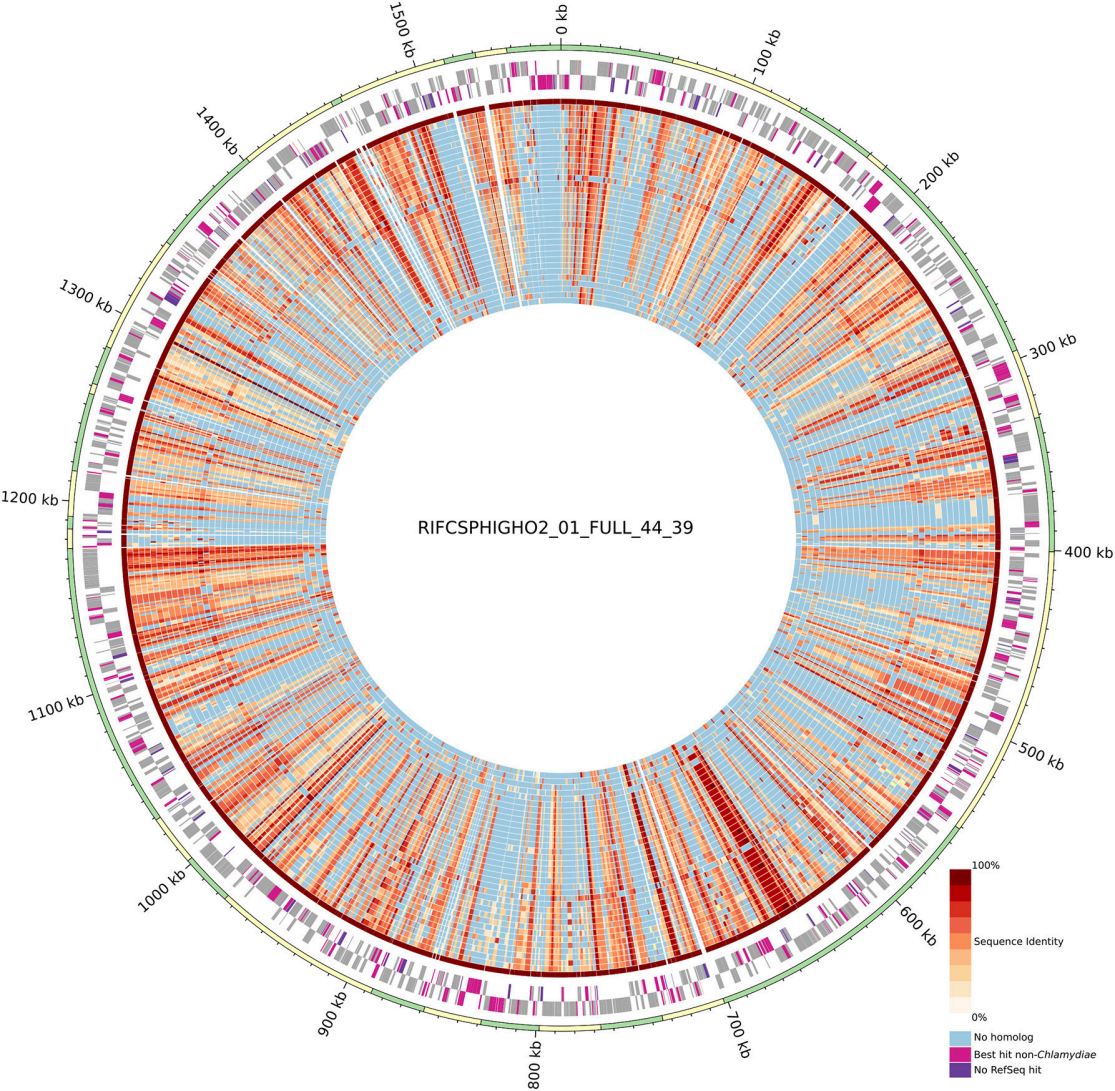
Circular representation of the uncultured genome RIFCSPHIGHO2_01_FULL_44_39 (accession: MGLR01000000). The outer circle indicates contig boundaries. The predicted open reading frames (ORFs) of the leading and lagging strands are reported in gray. Pink ORFs are proteins without a best hit against chlamydial sequences in the RefSeq database. Violet ORFs are proteins without any significant hit in the RefSeq database. The inner blue/red circles show the conservation of the closest identified orthologous protein (red scale) in the 37 other chlamydial species and one other genome of group 3 (Figure 1). Identity values were calculated based on the alignment of orthologous groups inferred using OrthoFinder. The absence of any ortholog is indicated in blue. Most contigs exhibit a majority of best PLAST hits against chlamydial sequences.

Despite the metagenomics approach to sequence *Chlamydiae* from more complex samples, only few genomes exhibited signs of contamination. The strongest evidence was present in the *S. epinepheli* genome where 32 of the 104 nearly universal proteins were present in more than one copy, indicating that it might be an admixture of two genomes (Figure 1C). Nevertheless, the 32 duplicated *S. epinepheli* markers were all monophyletic in phylogenetic reconstructions including all chlamydial homologs and their closest homologs in the RefSeq database, indicating that the assembly might be an admixture of two closely related strains. In order to further evaluate if the assemblies contained only fragments of *Chlamydiae* genomes, all CDS were compared to the RefSeq database. A majority of CDSs of each analyzed genome exhibited a best non-identity PLAST hit (excluding hits against RefSeq sequences classified as belonging to the same species) against chlamydial sequences present in the RefSeq database (Figure 1C). Assemblies reconstructed from metagenomics and single cell data exhibit between 55.77 and 75.93% of best PLAST hits against chlamydial sequences, which is higher than for *S. negevensis* (45.01%). *S. negevensis* is the only representative of the family *Simkaniaceae* currently available in RefSeq. The higher proportion of PLAST hits against non-chlamydial sequences (32.48%) and without any hits (22.51%) as compared to representative of other new families absent from RefSeq might be related to the much larger size of *S. negevensis* genome (Figure 1B). Several of the most fragmented assemblies harbored contigs without any best hits against chlamydial sequences (Figure 1C). Nevertheless, chlamydial hits were systematically distributed along the whole assembly, with only few small contigs exhibiting no best hits against chlamydial sequences, as can be seen on Figure 2 for the assembly RIFCSPHIGHO2_01_FULL_44_39. Overall, these results indicate that all assemblies contain mostly chlamydial DNA sequences, supporting the reliability of the analysis.

### Classification and diversity of new chlamydial genomes

**Figure 3:**
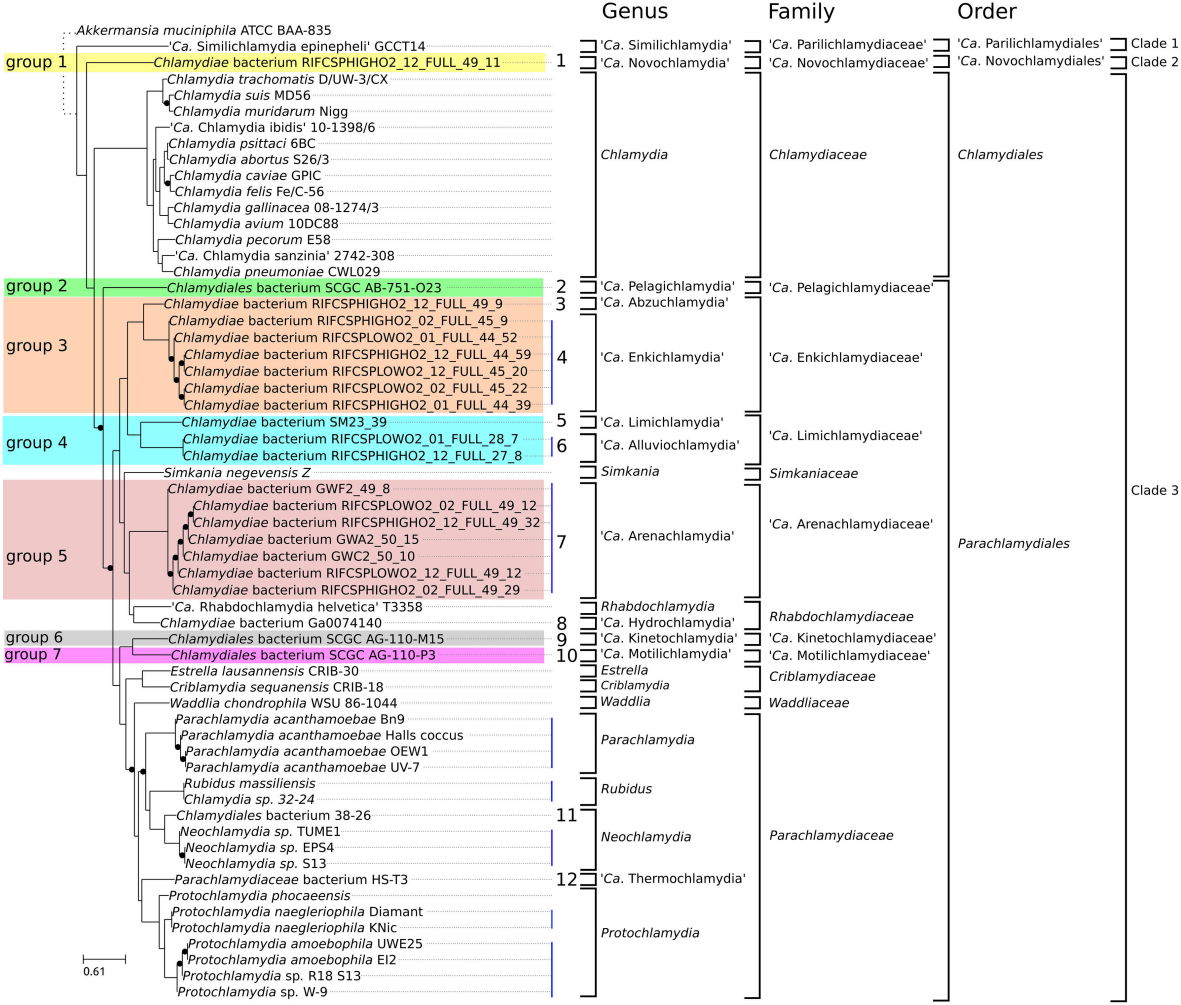
Updated classification of the order *Chlamydiales* based on genomic data. Proposed new *Candidatus* genera, families and orders are indicated in bold. Only three genera (*Chlamydia, Neochlamydia* and *Protochlamydia*) have more than one species. Vertical blue lines indicate genomes of the same species. Numbers from 1 to 12 indicate new candidate species. Candidate species were not named. Eleven new Candidatus genera are proposed. Novochlamydia (from Latin novus, new), Pelagichlamydia (from the Latin world pelagus, sea), Abzuchlamydia (from Abzu, the underground waters of the Aquifer in the Sumerian mythology), Enkichlamydia (from the Sumerian god Enki, lord of the Abzu), Limichlamydia (from the Latin world limus, silt, alluvium), Alluviochlamydia (from the Latin world alluvio, alluvium), Arenachlamydia (from the Latin world Arena, sand), Hydrochlamydia (from the Greek prefix hydro-, relating to water), Kinetochlamydia (from the Greek prefix kineto-, motion; movement), Motilichlamydia (from Latin world motus, past participle of movere, to move), Thermochlamydia (from the Greek world thermos, warm, hot).

We recently proposed a scheme for the classification of chlamydial genomes at various taxonomic levels (species, genus and family). This scheme was used here to update the classification of all unclassified chlamydial genomes (Figure 3). Briefly, the strain HS-T3 likely belongs to a new genus in the family *Parachlamydiaceae* (Figure S2). *Chlamydia* sp. 38–26 is a new *Neochlamydia* species (Figure S3) and *Chlamydia* sp. 32–24 is a *Rubidus massiliensis* strain (Figure S4). There are four *Pr. amoebophila* strains (Figure S5) and three strains of the same *Neochlamydia* species (S13, TUME1 and EPS4, Figure S6).

Regarding the new deeply branching assemblies, Group 3 and Group 4 encompass each two candidate species from two different genera (Figures S7, S8). The seven assemblies of group 5 are part of the same candidate species (Figure S9). Altogether, the new genomes can be classified into 12 new candidate species (Figure 3). Half of them are only represented by <90% complete genomic data (Figure 1B, Table 1).

Most genome assemblies sequenced using a metagenomics approach lack rRNA operons: 15 lack 16S rRNA, whereas 17 lack 23S rRNA (Figure S2). This happens frequently as rRNA operons are too highly conserved to be assigned reliably to specific genome assemblies. In addition, most genomes lack one or several of the nine proposed taxonomic markers (Figures S2–S9; Pillonel et al., 2015). This is a major limitation for any scheme relying on a limited set of genes. Therefore, in order to evaluate the genus and family level diversity of the *Chlamydiae* phylum, the identity of reciprocal best blast hits (RBBH) was used to evaluate the divergence of chlamydial lineages at various taxonomic depths.

**Figure 4:**
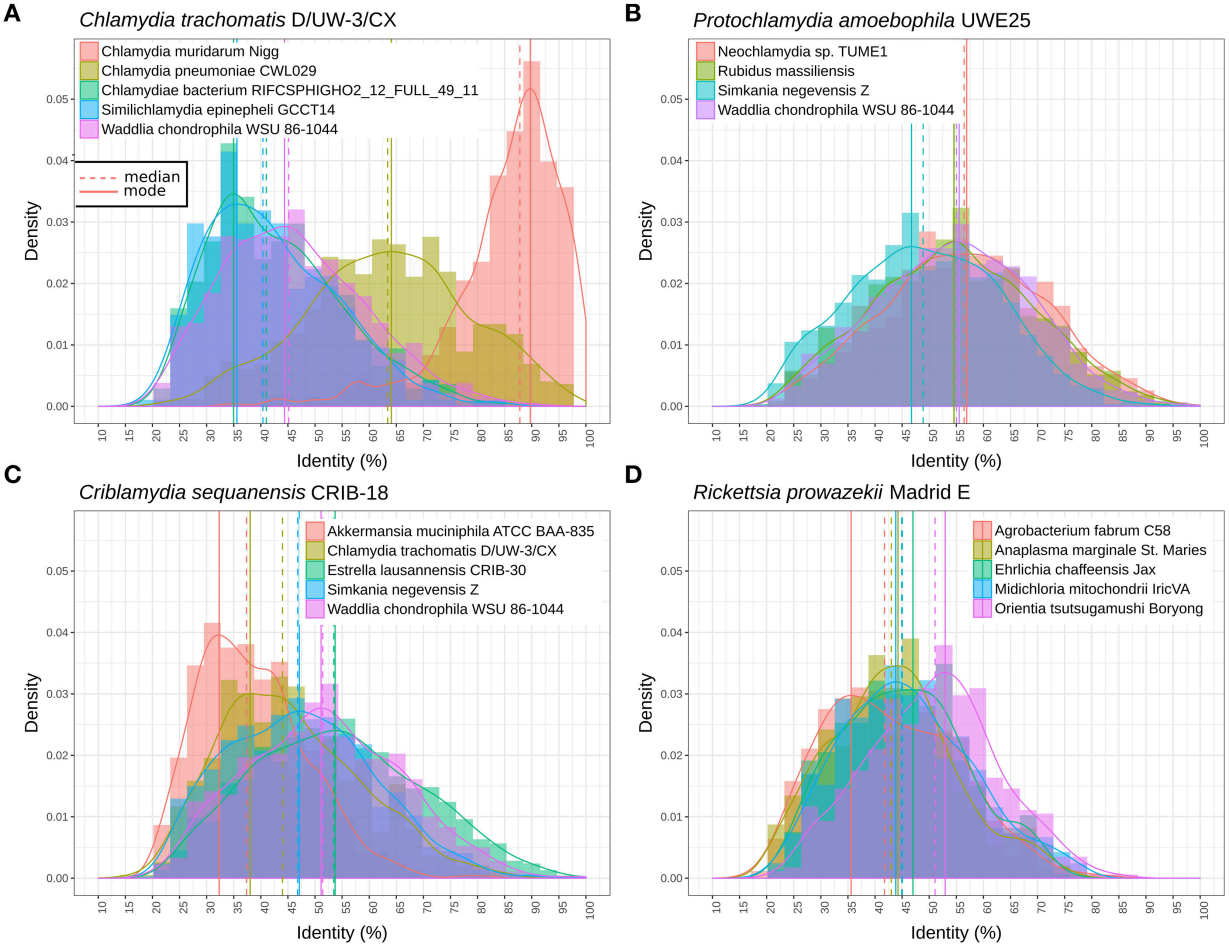
Distribution of the identity of reciprocal best blast hit between pairs of genomes. **(A)** Protein sequences exhibit similar levels of divergence between different *Parachlamydiaceae* genera and *Waddlia chondrophila* (different family), with a median amino acid identity around 55%. The distribution is clearly shifted for *Simkania negevensis*, with a median identity inferior to 50%. **(B)** Distribution of protein identities between *C. trachomatis* and the closely related *C. muridarum* genome, *C. pneumoniae, W. chondrophila*, and two deep branching taxa (*S. epinepheli* and one groundwater metagenomic bin). The continuous decline in sequence identity with divergence time is clearly visible here. The most distant chlamydial lineages are extremely divergent and skewed, with a lot of lowly conserved proteins and some highly conserved ones (mode of 35% sequence identity). **(C)** Distribution of sequence identities of *C. sequenanesis* and representatives of four recognized families and the outgroup, *A. muciniphila*. **(D)** Identical analyses comparing the obligate intracellular bacteria *Rickettsia prowazekii* (alpha-proteobacteria) with *Orientia tsutsugamushi* (*Rickettsieae*), *Anaplasma marginale* (*Anaplasmataceae*), *Ehrlichia chafeensis* (*Anaplasmataceae*), *Midichloria mitochondrii* (“*Candidatus* Midichloriaceae”), and *Agrobacterium fabrum* (free-living member of the *Rhizobiales* order).

Figure 4 shows the distribution of RBBH identity values between pairs of genomes. As expected, the distribution of sequence identity values shifts continuously toward lower values with increased phylogenetic distance (Figure 4A). *W. chondrophila* and the different genera classified in the *Parachlamydiaceae* family show similar levels of divergence (Figure 4B). This is not the case with *S. negevensis*, exhibiting a median identity lower than 50% with *P. amoebophila* (Figure 4B). The median of the median pairwise identities among members of the *Chlamydiales* and *Parachlamydiales* (Figure 3) is of 44.44%, whereas *Chlamydiaceae* and the two deep branching clades 1 and 2 exhibit a median identity of respectively, 39.81 and 41.14% (using one representative genome per species, see detailed values in Figure S10). The high divergence of clade 1 and 2 is even more obvious on Figure 4A, with a clear shift of the distribution of the RBBH identities peaking at about 35% identity between *C. trachomatis* and clade 1 and 2. Altogether, the phylum exhibits at least three highly divergent clades, and may now include seven new candidate family-level lineages and 11 new genera (Figure 3, Figure S11).

### Correlation between 16S rRNA sequence conservation and whole genome relatedness

Gupta and colleagues recently disputed the 16S rRNA identity cutoffs proposed to delineate families of the phylum *Chlamydiae*, arguing that the number of chlamydial families was inflated by the high cutoff in use (Gupta et al., 2015). Nevertheless, different bacterial clades may exhibit variable rates of sequences evolution (Kuo and Ochman, 2009). In addition, the 16S rRNA gene may not evolve at a constant rate across the entire tree of life and may not necessarily be a reliable indicator of whole genome relatedness (Konstantinidis and Tiedje, 2005; Kuo and Ochman, 2009). In order to put the *Chlamydiae* phylum into a broader perspective, we compared the 16S rRNA pairwise sequence identity and the conservation of protein sequences with data from another diverse group of intracellular bacteria, the order *Rickettsiales*. Representatives of the *Rhizobiales* order were also included for comparison (Table S5).

**Figure 5:**
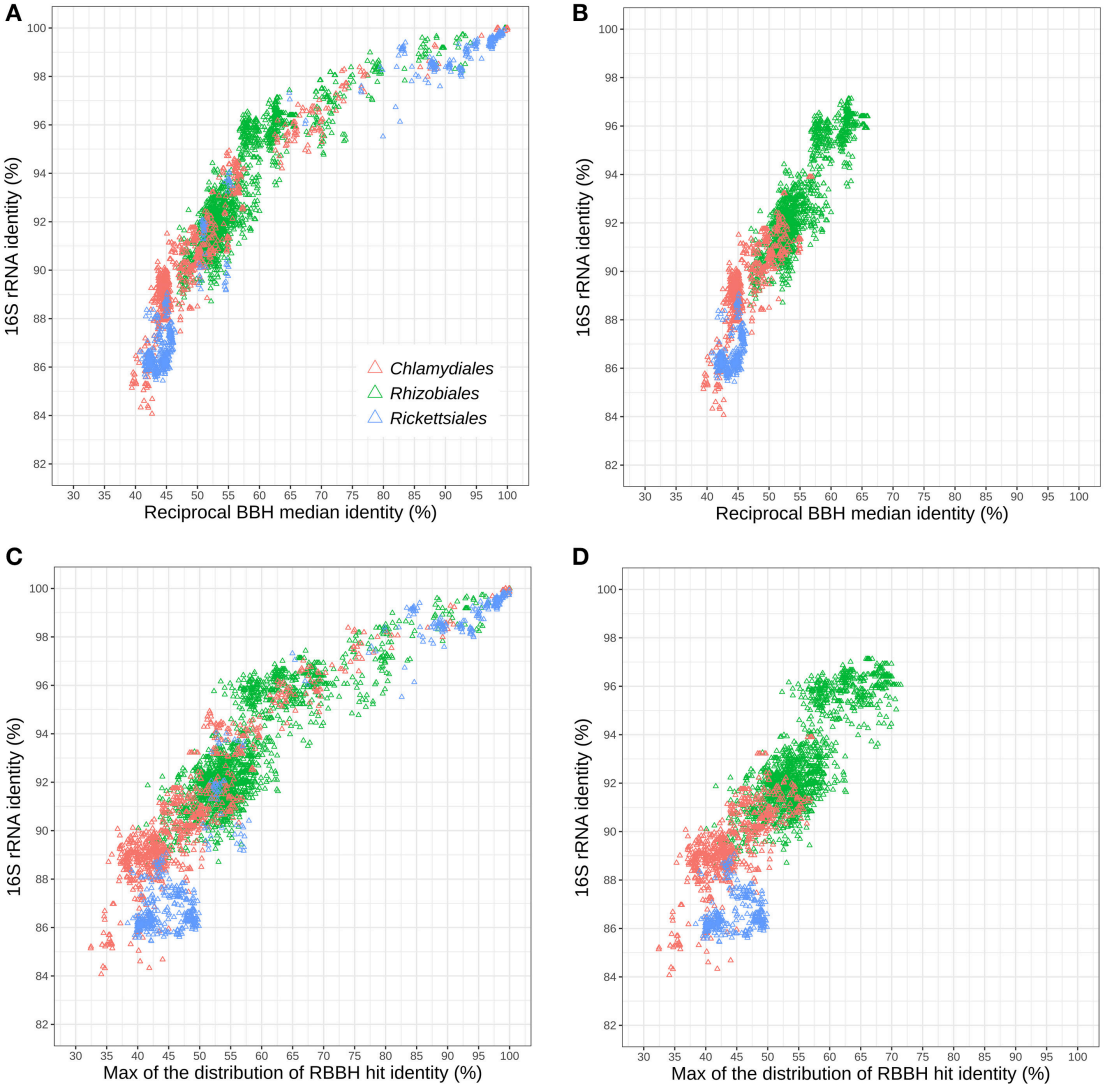
Relationships between 16S rRNA and protein sequences conservation within three distinct bacterial orders: *Rhizobiales, Chlamydiales*, and *Rickettsiales* (indicated with colors). **(A)** Relationships between 16S rRNA identity and the median protein identity within the three bacterial orders and **(B)** for different families of the same order. **(C)** Relationships between 16S rRNA and the modal identity within the three bacterial orders and **(D)** between different families of the same order.

Figure 5A shows the relationships of 16S rRNA and median RBBH identities for the three considered orders. For both measures, the *Chlamydiales* and *Rickettsiales* orders exhibit higher sequence divergence than the *Rhizobiales* order. The sequence divergence of different families belonging to the same order (see Tables S5, S6 for the detailed classification used here) reveals that the 16S rRNA gene of *Rickettsiales* families is more divergent than in the *Chlamydiales* families (Figure 5B). On the other hand, the median pairwise protein identity is also relatively low for most *Chlamydiales* (Figures S12A–D). Given the skewed shape of RBBH identity distributions (Figure 4), the median identity may not be the most appropriate summary metrics to estimate genome divergence. An alternative would be the maximum of the density distribution of RBBH identity rather than the median (Figures 5C,D). Using the maximum of the estimated distribution of amino acid identities, the *Chlamydiae* cloud shifted toward lower values, indicating that the distribution of RBBH identities in pairs of chlamydial genomes is more skewed than for *Rickettsiales* and *Rhizobiales*. This still holds true when comparing *Parachlamydiales* only (excluding *Chlamydiaceae* and *Similichlamydiaceae*).

The difference between *Rickettsiales* and *Chlamydiales* could be due to the fact that the *Chlamydiales* dataset includes incomplete genomes. Missing data might lead to spurious reciprocal best blast hits, leading to an undervaluation of whole genome relatedness. Nevertheless, the trend is still visible when only complete chlamydial genomes are considered (Figures S12E,F). In conclusion, chlamydial families exhibit higher conservation of 16S rRNA but a skewed distribution of whole genome RBBH identity toward values that are as low, or even lower than genomes exhibiting lower 16S rRNA identities.

### Conservation of mechanisms involved in the interaction with eukaryotic hosts and other microbial cells

**Figure 6:**
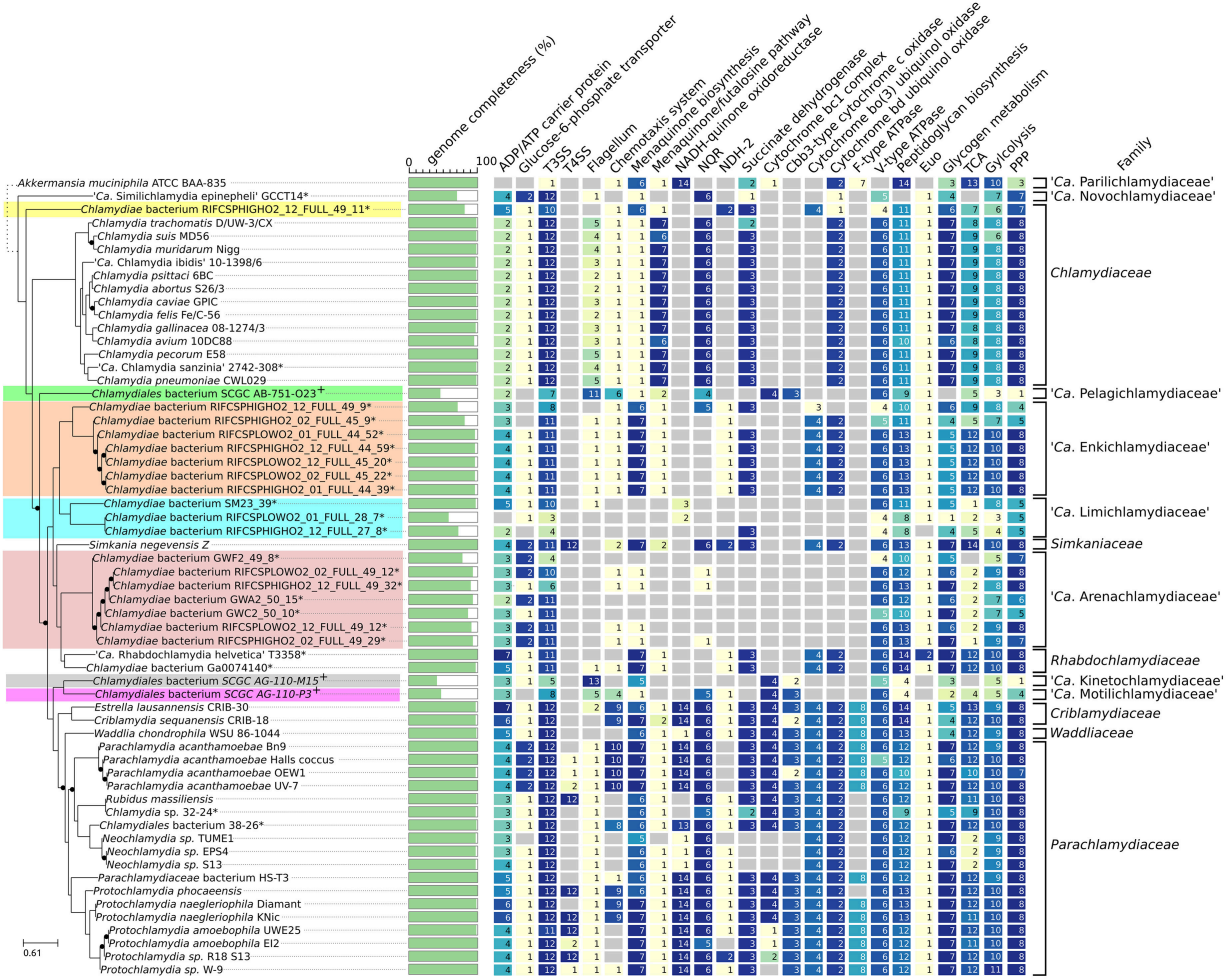
Comparative genomics of the phylum *Chlamydiae*. Identified homologs of proteins involved in nucleotide uptake (ntt), part of molecular machines (i.e., T3SS, Flagellum) and part of selected metabolic pathway. For KEGG pathways/modules, number indicates non redundant count of KEGG orthologs part of the corresponding pathway/module. Reference locus tag, KEGG and Pfam accessions are indicated in Table S1.

Several genomes included in this analysis are only a partial representation of the true chlamydial genome, which precludes any in-depth genome content comparisons. Nevertheless, the identification of even partial molecular machineries and metabolic pathways can provide first insights into the ancestrality and conservation of genomic features that may be essential to their unique lifestyle. The recent sequencing of *S. epinepheli*, the most diverging *Chlamydiae* described thus far, revealed the presence of a type III secretion system (T3SS) and the conservation of several key virulence factors (Taylor-Brown et al., 2017). T3SS components were identified in all new chlamydial clades (Figure 6, column T3SS), including the most incomplete ones. Several described T3SS effectors such as Mip and Nue are also widely distributed within the whole phylum (Figure S13). The entire clade that includes the *Simkaniaceae*, the *Rhabdochlamydiaceae* and several new lineages lack any homolog of Type II secretion system effector protease CPAF (Figure S13). In *C. trachomatis*, CPAF mutants display impaired generation of infectious elementary bodies (Snavely et al., 2014) and *cpaf* is therefore an essential gene. Our observations suggest that CPAF is dispensable in some chlamydial lineages, and its role in other *Chlamydia-*related organisms where it is conserved remains to be confirmed.

As obligate intracellular bacteria, *Chlamydiae* acquire essential nutrients from the cytosol of their host, including nucleotides and sugars such as ATP and glucose-6-phosphate. All but one partial genome encode at least two ADP/ATP antiporter homologs. These transporters are involved in nucleotide, ATP or nicotinamide adenine dinucleotide uptake from the host (Tjaden et al., 1999; Greub and Raoult, 2003; Haferkamp et al., 2004; Fisher et al., 2013). Furthermore, most genomes also encode a homolog of the glucose-6-phosphate transporter UhpC.

The genomes of *Simkania negevensis* and several *Parachlamydiaceae* strains encode an apparently complete type IV secretion system (T4SS) located either on the chromosome or on a plasmid (Bertelli et al., 2016) that may be involved in conjugative DNA transfer (Greub et al., 2004). Several strains of *Parachlamydia acanthamoebae* encode remnants of the T4SS in their genome (Greub et al., 2009; Bertelli et al., 2016). No traces of T4SS genes could be found in any other chlamydial lineages (Figure 6). The T4SS of *S. negevensis, Protochlamydia naegleriophila* KNic and *R. massiliensis* are located on a plasmid, which may have facilitated its loss or acquisition in the various chlamydial lineages. Assignment of plasmid sequences to specific organisms within the context of a metagenomic dataset is extremely difficult. Chlamydial plasmids may thus be missing in some assemblies, including for the genome of *Chlamydia* sp. 32–34.

It was recently suggested that some marine *Chlamydiae* may possess a flagellum regulated by a chemotaxis system (Collingro et al., 2017). The identification of several flagellar and chemotaxis genes in highly divergent chlamydial genomes retrieved by single cell-genomics was interpreted as an evidence that motility was an ancient feature of the *Chlamydiae* phylum (Collingro et al., 2017). Although most new uncultivated chlamydial clades were also sequenced from aquatic samples, none exhibited homologs of the flagellar genes identified in the genome assemblies SCGC AG-110-P3, AB-751-O23 and AG-110-M15 (Figure 6, group 2, 6, and 7), questioning whether this flagellar system is as old as Collingro et al. hypothesized. Two proteins of the type III secretion system that are homologous to flagellar proteins, fliQ/sctS (K02420) and fliN/sctQ (K02417), were sometimes annotated as part of the flagellum (Figure 6) but did not branch with SCGC *Chlamydiae* homologs in phylogenetic reconstructions (see for example Figure S14). A chemotaxis system is also encoded in several chlamydial genomes lacking flagellar genes including several *Parachlamydiaceae* (*P. phocaeensis, P. naegleriophila*, and *P. acanthamoebae*) and the two *Criblamydiaceae* genomes currently available. This system was thus proposed to be involved in signal transduction (Collingro et al., 2011). In the present analysis, chemotaxis genes were identified in the genome of *Chlamydia* sp. 38–26, which is closely related to *Neochlamydia* sp. strains that do not harbor a chemotaxis system. Overall, our data suggests that all *Chlamydiae* exhibit a similar obligate intracellular lifestyle, and that most are non-motile.

### Conservation of division mechanisms across the phylum *Chlamydiae*

All described *Chlamydiae* exhibit a similar biphasic developmental cycle, including the most deeply branching clades (Abdelrahman and Belland, 2005; Steigen et al., 2013; Seth-Smith et al., 2017). The transcription factor Euo targets more than 100 promoters in *W. chondrophila*, and may be a key player in cell cycle regulation (Domman and Horn, 2015; de Barsy et al., 2016). It is conserved throughout the whole phylum, including *S. epinepheli* and “*Ca*. Novochlamydiaceae” (Figure 6), with exception of three of the most incomplete genomes probably due to the partial data available. Interestingly, the genome assembly of *S. epinepheli* lacks all proteins involved in peptidoglycan biosynthesis (Figure 6) and all but three proteins involved in chlamydial division (Figure S13). Remnants of those proteins could not be found in the raw assembly using tBLASTn. Although the genome of this strain is partially covered, peptidoglycan synthesis genes are located in various regions along the chromosome, which reinforces the likely absence of these genes from *S. epinepheli*. Indeed, this bacterium divides through a budding process (Seth-Smith et al., 2017), which suggests that the family *Parilichlamydiaceae* may present a significantly different division mechanism than other chlamydial lineages.

### High metabolic diversity of the *Chlamydiae* phylum

Menaquinones are electron shuttles that allow the transfer of electrons between membrane-bound protein complexes in the electron transport chain (Meganathan, 2001). The *Chlamydiaceae* synthesize menaquinone through the alternative futalosine pathway (Hiratsuka et al., 2008; Barta et al., 2014). Other clades, including the deep branching “*Ca*. Novochlamydiaceae” (Figure 6), encode the traditional pathway, supporting the hypothesis that the futalosine pathway was horizontally acquired by the *Chlamydiaceae* lineage (Barta et al., 2014; Subtil et al., 2014). No menaquinone biosynthesis enzymes could be identified in the *S. epinepheli* genome (Figure 6). A homolog of MenB was identified (Figure 6) but it exhibits more similarity with enoyl-CoA hydratase and did not cluster with other chlamydial sequences in phylogenetic reconstructions, thus likely having another enzymatic role than MenB (Figure S15). Most genomes encode the necessary components of a minimal respiratory chain except the seven genomes of “*Ca*. Arenachlamydiaceae” (Figure 6). Those 1.4 Mb genomes are predicted to be nearly complete but do not encode the menaquinone biosynthesis pathway, nor any component of the respiratory chain, suggesting that they use alternative respiration systems.

Overall, enzymes of the respiratory chain exhibit variable patterns of parallel losses across the entire phylum. The NADH-quinone oxidoreductase (complex I) is for instance restricted to the *Parachlamydiaceae*-*Waddliaceae*-*Criblamydiaceae* clade, and was lost at least three times independently in subclades (Figure 6). Few proteins homologous to complex I were identified in the low GC genomes recovered from estuary sediments and groundwater (“*Ca*. Limichlamydiaceae”). They encode a putative NADP-reducing hydrogenase exhibiting 44.5–56% amino acid identity to the hnd operon of *Desulfovibrio fructosivorans* (Malki et al., 1995; Figure S16). The F-type ATPase is also restricted to *Parachlamydiaceae*-*Waddliaceae*-*Criblamydiaceae* clade, as opposed to the V-type ATPase that is conserved in all sequenced chlamydial genomes.

The citrate cycle (TCA) is incomplete in all *Chlamydiaceae* due to the absence of three enzymes; citrate synthase (GltA), aconitase (Acn) and isocitrate dehydrogenase (Icd) (Omsland et al., 2014). Several *Chlamydiae* such as the three *Neochlamydia* sp. and the seven “*Ca*. Arenachlamydiaceae” genomes (repeatedly missing the same set of enzymes) exhibit an even less complete TCA (Figure 6). Indeed, no homologs of TCA enzymes could be identified in the *S. epinepheli* genome assembly. On the other hand, several homologs of the pentose phosphate pathway (PPP), glycolysis and glycogen metabolism were identified in all assemblies that are more than 90% complete (Figure 6).

The various *Parachlamydiaceae* species exhibit significant differences in their ability to synthetize amino acids, with reduced biosynthetic abilities in *Protochlamydia amoebophila* strains and *Neochlamydia* strains and extended metabolic abilities for *Parachlamydia* strains (Figure S13). The 3 closely related *Neochlamydia* genomes exhibit genomes larger than 2.5 Mbp, but overall highly reduced metabolic capacities (Figures S17, S18). On the other hand, they encode large numbers of mobile genetic elements and repeat proteins (Figure S17). *S. epinepheli* has the most reduced metabolic capacities, but its genome is only about 70% complete. While the metabolism of amino acids, nucleotides, cofactors and vitamins was not investigated in detail, we generally observed that all genomes had limited predicted biosynthetic capabilities for all those compounds and that marked differences exist within the phylum (Figure S13), as already observed in previous comparative analyses (Bertelli et al., 2010, 2015; Collingro et al., 2011; Omsland et al., 2014).

## Discussion

Shotgun metagenomics allows the reconstruction of genomes from complex microbial communities. It can yield draft and nearly complete genomes without the need for cultivation (Alneberg et al., 2014), allowing to investigate the biology of uncultivable or difficult-to-culture organisms, including members of the phylum *Chlamydiae*. The comparative analysis of 21 metagenomic bins from public databases with previously described genomes allowed the identification of seven new candidate family level lineages, 11 new candidate genera and 12 new candidate species (Figure 3). Six of those new species exhibit nearly complete genomes (more than 90 % complete).

A reference phylogeny was reconstructed based on 108 single copy orthologs conserved in more than 90% of the genomes. Given the incomplete nature of the dataset and the high sequence composition bias of several genomes, the reconstructed phylogeny might not precisely reflect the true evolutionary relationships between the most deeply branching lineages. Nevertheless, this phylogeny is congruent with recently published phylogenies based on 16S rRNA and whole genome datasets (Figure 1; Gupta et al., 2015; Pillonel et al., 2015). While no information regarding host or lifestyle is available for the newly identified clades, genomic analyses support the hypothesis that they all share the same obligate intracellular lifestyle. They all encode a type III secretion system, homologs of known effector proteins and transporters involved in nutrient uptake. In addition, members of the *Parilichlamydiaceae*, the most deeply branching lineage in the current phylogeny, are fish pathogens that share the typical biphasic life cycle of other *Chlamydiae* (Steigen et al., 2013; Stride et al., 2013; Seth-Smith et al., 2017). Despite these similarities, some central mechanisms such as cell division might significantly differ in distantly-related clades such as *S. epinepheli*. No homologs of most proteins involved in peptidoglycan biosynthesis and cell division could be identified in the *S. epinepheli* assembly. This partial genome also shows particularly limited capacities for the synthesis of amino acids, effectors, vitamins and nucleotides. Pairwise protein sequences comparisons revealed that *S. epinepheli* is highly divergent as compared to previously sequenced members of the phylum. This high sequence divergence might impair the correct identification of homologs based on amino acid sequence comparisons. The discovery and sequencing of novel intermediate species should facilitate the identification of groups of orthologs among such distantly-related organisms. It should clarify the origin of these highly different biosynthetic abilities and further differences in core chlamydial mechanisms.

Several genomes predicted to be nearly complete showed little evidences of autonomous energy production capabilities. Those *Chlamydiae* might completely depend on their host for energy generation. Protein complexes of the respiratory chain are expressed at the end of the replication cycle (König et al., 2017). They might be essential at the extracellular stage. Chlamydial lineages which have lost the complete respiratory chain might not present the classical biphasic life-cycle of most *Chlamydiae*. Elementary bodies (EB) and cell lysis were never described for any of the three *Acanthamoeba* endosymbionts of the *Neochlamydia* genus (Ishida et al., 2014). The transition from a biphasic life-style to vertical transmission is expected to lead to major changes at the genomic level. Those three strains exhibit genomes more than twice larger than *Chlamydiaceae* spp., but highly reduced functional repertoires as compared to other *Parachlamydiaceae* (Figures S17, S18). They also harbor a high number of mobile genetic elements and repetitive elements (Figure S17; Domman et al., 2014). Similar genome features were observed in recently established insect endosymbionts such as *Serratia symbiotica* and *Sodalis glossinidius* (Toh et al., 2006; Lamelas et al., 2011). Gene losses strongly affect carbohydrate biosynthetic pathways and the production of energy, as illustrated by the loss of multiple components of the respiratory chain (Figure 6). The reduction of the electron transport chain is associated with the near complete loss of the tricarboxylic acid (TCA) cycle in at least two distinct lineages (“*Ca*. Arenachlamydiaceae” and the three *Neochlamydia* spp.).

The loss of glycogen metabolism is generally associated with parasitic bacteria (Henrissat et al., 2002). This is not the case for *Chlamydiae* (Omsland et al., 2014). Indeed, homologs of the enzymes involved in the biosynthesis of glycogen were identified in all new assemblies. One hypothesis to explain the conservation of glycogen biosynthesis in these obligate intracellular organisms is that these enzymes are effectors manipulating the host metabolism (Ball et al., 2013; Ball and Greub, 2015).

Two-fold variations in genome GC content can be observed across the phylum. The genomes of two related species of “*Ca*. Limichlamydiaceae” exhibit a GC content lower than 30%, similarly to some primary and secondary insects symbionts such as *Buchnera* and *Spiroplasma* (Moran et al., 2008; Lo et al., 2016). The increasing genomic coverage of the phylum, including the small low GC genome clades and five different genera of the *Parachlamydiaceae*, indicates that gene losses occur in parallel in different chlamydial lineages, and that highly reduced genomes are not restricted to the *Chlamydiaceae* family. Nevertheless, additional complete genome sequences will be necessary to gain further knowledge on the variable patterns of genome evolution across the phylum.

Family-level lineages of the phylum *Chlamydiae* exhibit a higher conservation of the 16S rRNA gene as compared to members of the *Rickettsiales* order. Yet, the comparison of complete and nearly complete genomes suggest that chlamydial protein sequences show similar level or increased level of divergence for the most distantly related families. The distribution of RBBH identities seems to be more skewed toward lower values in the phylum *Chlamydiae*. Endosymbiosis is characterized by an acceleration of the rate of evolution (Itoh et al., 2002; Woolfit and Bromham, 2003; Kuo and Ochman, 2009). The *Buchnera* synonymous rate of evolution was for instance evaluated to be about twice that of low-codon-bias genes of *Escherichia coli* and *Salmonella typhimurium* (Clark et al., 1999). This is particularly due to an accelerated accumulation of mutations by genetic drift due to the small effective population size of those bacteria (Moran, 1996), but also to the loss of DNA repair mechanisms (Moran and Bennett, 2014). Members of the *Chlamydia* genus lack homologs of several repair proteins such as MutT, MutM, and MutH (Stephens et al., 1998). Several attempts were made to evaluate the mutation rate and the impact of genetic drift on members of the *Chlamydiaceae* family. They report a rather high dN/dS ratio for *C. trachomatis* and *C. pneumoniae*, which is indicative of increased level of genetic drift (Rocha et al., 2006; Kuo et al., 2009; Joseph et al., 2012). Estimates of mutation rates differ by several folds between studies, and were recently estimated to be similar to free-living bacteria for *C. trachomatis* (Joseph et al., 2012; Hadfield et al., 2017). As several genomes are now available for several *Chlamydia*-related species, it would be interesting to estimate and compare the dN/dS ratio of other clades.

The 16S rRNA, a gene under high purifying selection, is more conserved than protein sequences in the phylum *Chlamydiae*. This difference is larger in the phylum *Chlamydiae* than in other intracellular bacteria such as the *Rickettsiales* and might indicate that the accelerated rate of evolution of *Chlamydiae* is at least partly due to an increase in mutation rate rather than genetic drift alone. Drift might also be counter balanced by the strong selection pressure on rRNA (Woolfit and Bromham, 2003). Those parameters might nevertheless vary within the phylum itself. Members of the *Parachlamydiales*, which infect free-living amoebae, exhibit larger genomes and are expected to be less sensitive to genetic drift owing to their larger effective population size and reduced transmission bottlenecks as compared to vertebrate parasites of the *Chlamydia* and *Similichlamydia* genera. The skewed identity distribution is indeed more accentuated when members of the *Chlamydiaceae* and *S. epinepheli* are included in the comparison, two clades exhibiting genomes of highly reduced size. Considering the relative high divergence of *Chlamydiae* genomes as compared to the 16S rRNA sequence, dedicated cutoffs are needed to properly interpret 16S rRNA based surveys of chlamydial diversity. Such cutoffs specific for members of the *Chlamydiae* phylum have been previously proposed (Pillonel et al., 2015).

Gupta and colleagues recently proposed to split the phylum *Chlamydiae* into the order *Chlamydiales* and *Parachlamydiales* (Figure 3; Gupta et al., 2015). They also recognized the existence of the deep branching “*Ca*. Parilichlamydiaceae” clade, but included it in the *Parachlamydiales* order. The current work confirms the high level of divergence of the “*Ca*. Parilichlamydiaceae,” in addition to a potential new deeply branching lineage (“*Ca*. Novochlamydiaceae”). In addition, they appear to exhibit fundamental differences in division mechanisms that underline the need to create additional orders in the taxonomical classification for those deep branching lineages. The data presented is this study support the division of the *Chlamydiae* phylum into four orders: the *Parachlamydiales, Chlamydiales*, “*Candidatus* Parilichlamydiales” and “*Candidatus* Novochlamydiales.” While much work is still needed to fully comprehend the biology and diversity of members of the phylum *Chlamydiae*, this work provides a first insight into the metabolic and genetic diversity of what could be the most ancient and diverse clade of intracellular parasites of eukaryotes.

## Author contributions

TP designed the study, performed the analyses and wrote the manuscript. TP and CB contributed to the interpretation of the results and the redaction of the manuscript. GG contributed to the design of the study and the redaction of the manuscript.

### Conflict of interest statement

The authors declare that the research was conducted in the absence of any commercial or financial relationships that could be construed as a potential conflict of interest.

## Supplementary material

Figure S1 Evolution of the pan and core-genome size of the phylum *Chlamydiae*. **(A)** Evolution of the size of the pan and core-genome with the addition of new genomes (based on 10 random sampling of genomes up to the total of genomes available). **(B)** Barplot showing the number of orthogroups identified in one to the 59 genomes included in the analysis. A large majority of orthologous groups are restricted to one or only few genomes.

Figure S2 Percentage of nucleotide and amino acid identitiy of the nine taxonomic markers as compared to *Parachlamydiaceae* sp. HS-T3. Sequence conservation suggest that strains with green rows are part of the same family (*Parachlamydiaceae*). The low conservation of DnaA, SucA, protein 325 and FabI supports classification of *Parachlamydiaceae* sp. HS T3 as the unique representative of a new *Parachlamydiaceae* genus.

Figure S3 Percentage of nucleotide and amino acid identitiy of the nine taxonomic markers as compared to *Chlamydiales* sp. 38 26. The 16S and 23S rRNA sequences were missing from the metagenomic bin, but the relatively high conservation of DnaA, SucA, protein 325 and FabI and low conservation of species-level markers (RpoN, FtsK, PepF, Adk, and HemL) suggest that the *Chlamydiales* sp. 38 26 is a new species of the genus *Neochlamydia*.

Figure S4 Percentage of nucleotide and amino acid identity of the nine taxonomic markers as compared to *Rubidus massiliensis*. Sequence conservation of both genus and species-level taxonomic markers indicate that *Chlamydia* sp. 32 24 is a new *R. massiliensis* strain.

Figure S5 Percentage of nucleotide and amino acid identity of the nine taxonomic markers as compared to *Protochlamydia amoebophila*. Sequence conservation of DnaA, SucA, protein 325 and FabI indicates that *Protochlamydia* sp. R18 S13 and *Protochlamydia* sp. W 9 are *P. amoebophila* strains.

Figure S6 Percentage of nucleotide and amino acid identity of the nine taxonomic markers as compared to *Neochlamydia* sp. TUME1. Sequence conservation of both genus and species-level taxonomic markers indicate that the three *Neochlamydia* assemblies are representatives of the same species.

Figure S7 Percentage of nucleotide and amino acid identity of the nine taxonomic markers as compared to Group 3 (“*Ca*. Enkichlamydiaceae”). The conservation of RpoN, PepF, Adk, and HemL indicates that all six assemblies of group 3 (“*Ca*. Enkichlamydiaceae”) are part of the same species. The conservation of protein 325 and FabI provide contradictory results regarding the classification of *Chlamydiae* sp. RIFCSPHIGHO2 12 FULL 49 9 as part of the same genus or not.

Figure S8 Percentage of nucleotide and amino acid identity of the nine taxonomic markers as compared to *Chlamydiae* bacterium SM23 39. The conservation of DnaA, protein 325 and FabI indicate that group 4 (“*Ca*. Limichlamydiaceae”) can be subdivided in two genera.

Figure S9 Percentage of nucleotide and amino acid identity of the nine taxonomic markers as compared to Group 5. The high level of conservation of RpoN, FtsK, and PepF indicates that all 7 group 5 (“*Ca*. Arenachlamydiaceae”) assemblies are part of the same species. The absence of 16S and 23S rRNA sequences precludes family-level classification based on the published scheme.

Figure S10 RBBH identity distribution of newly identified clades. **(A)** The two clades of marine Chlamydiae exhibit a median pairwise RBBH identity lower than 50%. Of note, those genomes are <50% complete. *S. negevensis* and *W. chondrohila* were added for comparison. **(B)** Comparison of representatives of the two genera of group 3. *S. negevensis* and *Chlamydia* sp. SM 23–39 were added for comparison. **(C)** The divergence of the two putative genus of group 4 is nearly as important as with other families **(D)** *Chlamydia* sp. 28–36 most closely related to *Neochlamydia* sp. **(E)** Comparison of the density distribution for different species of the *Protochlamydia* genus and comparison with representatives if two other genus. **(F)** The *Parachlamydia* sp. HS-T3 shows high sequence divergence (median RBBH identity of about 55%) with representatives of other *Parachlamydiaceae* genus.

Figure S11 Median RBBH identity heat map. Median identity of reciprocal best blast hits calculated between one representative of each species and Candidatus species of the Phylum *Chlamydiae*.

Figure S12 Density median RBBH and modal identity between families and orders, and complete chlamydia genomes comparisons. For this comparison, only a single order for the phylum *Chlamydiae* was considered. **(A)** Distribution of the median identity between families within three different orders. **(B)** Distribution of the median identity between families within three different orders. **(C)** Distribution of the median identity between the *Rhizobiales* and *Rickettsiales* orders (alpha-proteobacteria). **(D)** Distribution of the modal identity between the *Rhizobiales* and *Rickettsiales* orders (alpha-proteobacteria). **(E)** The relationship between 16S rRNA sequence identity and the median RBBH identity between families within three distinct bacterial orders. Only complete genomes were included. **(F)** The relationship between 16S rRNA sequence identity and the modal RBBH identity between families within three distinct bacterial orders. Only complete genomes were included.

Figure S13 Identified homologs of effectors/inclusion membrane proteins, Membrane, and division proteins, as well as number of homologs identified in selected biosynthesis pathways. Data based on GhostKOALA annotation and clustering into orthologous groups with Orthofinder. Reference locus tags and KEGG accessions are indicated in Table S1.

Figure S14 Phylogeny of the EscS/YscS/HrcS family type III secretion system export apparatus protein. The phylogeny include the 2 closest RefSeq and SwissProt hits of each chlamydial sequence. Amino acid sequences were aligned with mafft version 7.058b and the phylogeny was reconstructed using Fasttree 2.1.9 with default parameters.

Figure S15 Phylogeny of the orthologous group including menB. The phylogeny includes the 2 best UniProt and 4 best RefSeq hits of each sequence of each protein of the orthologous groups. The menB homolog of *Chlamydiae* and their homologs in the genomes of *S. epinepheli* are not monophyletic. Amino acid sequences were aligned with mafft version 7.058b and the phylogeny was reconstructed using Fasttree 2.1.9 with default parameters.

Figure S16 Phylogeny of the orthologous group including NADH-quinone oxidoreductase subunit E. The phylogeny includes the 2 best UniProt and 4 best RefSeq hits of each sequence of the orthologous groups. The NADH-quinone oxidoreductase subunit E orthologs from *Chlamydiae* and their homologs in low GC chlamydial genomes are not monophyletic. Amino acid sequences were aligned with mafft version 7.058b and the phylogeny was reconstructed using Fasttree 2.1.9 with default parameters.

Figure S17 Classification of protein into COG categories. The red gradient reflect the number of annotated proteins with the corresponding COG category and was normalized for each column (each COG category). The three *Neochlamydia* spp. encode a relatively large number of CDS (1,879–2,232), but a reduction in most COG categories except for mobile genetic elements (X) and transcription (K) as compared to closely related *Parachlamydiaceae*. The large increase in the transcription category in the 3 *Neochlamydia* genomes is due to the presence of a very large number of Leucine-rich repeat (LRR) proteins.

Figure S18 Comparison of KEGG functional categories for the Neochlamydia strains and their closest phylogenetic neigbour. Counts indicates the non-redundant number of Kegg Orthologs (KO) identified for each functional category. Gene losses are particularly important in categories such as ATP synthesis, amino acid metabolism and carbohydrate metabolism.

Table S1 Reference locus, KEGG pathways and modules found in Figure 1, and Figure S13.

Table S2 Complete list of OrthoFinder orthologous groups (one group per row). CDS are identified with Genbank or RefSeq locus tags.

Table S3 List of core orthologous groups conserved in 90% of the genomes (55 out of 60 genomes). The RefSeq/Genbank locus tags of the CDS of each orthologous groups can be found in Table S2.

Table S4 Detailed checkM results.

Table S5 List and detailed taxonomy of *Rickettsiales* and *Rizhobiales* genomes used in this study.

Table S6 Detailed *Chlamydiales* classification used in this study.
