# Supplementary material for: Environmental Metagenomic Assemblies Reveal Seven New Highly Divergent Chlamydial Lineages and Hallmarks of a Conserved Intracellular Lifestyle
Source: Front Microbiol. 2018 Feb 20;9:79. doi: 10.3389/fmicb.2018.00079 (PMC5826181; doi:10.3389/fmicb.2018.00079)
Supplement: Figure S1 — Evolution of the pan and core-genome size of the phylum Chlamydiae. (A) Evolution of the size of the pan and core-genome with the addition of new genomes (based on 10 random sampling of genomes up to the total of genomes available). (B) Barplot showing the number of orthogroups identified in one to the 59 genomes included in the analysis. A large majority of orthologous groups are restricted to one or only few genomes. [file Image1.pdf]

Supplementary data: Environmental metagenomic assemblies reveal seven new highly divergent chlamydial lineages and hallmarks of a conserved intracellular lifestyle

Trestan Pillonel[1], Claire Bertelli[1], and Gilbert Greub[1]

[1]Center for Research on Intracellular Bacteria, Institute of Microbiology, University Hospital Center and University of Lausanne, Switzerland

November 2017

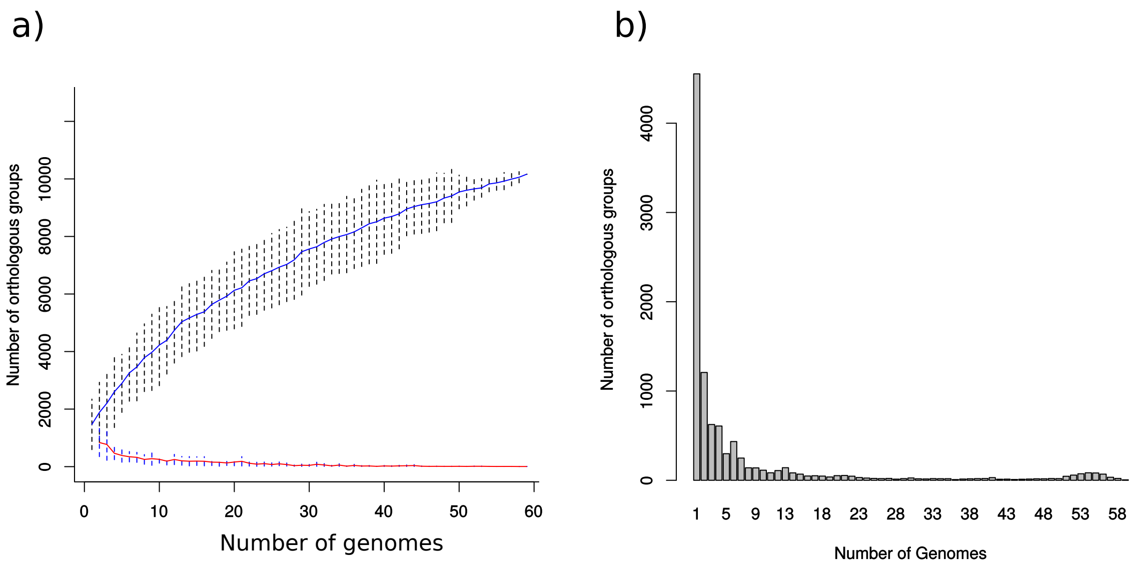

Figure S1: Evolution of the pan and core-genome size of the phylum *Chlamydiae*. **a**) Evolution of the size of the pan and core-genome with the addition of new genomes (based on 10 random sampling of genomes up to the total of genomes available). **b**) Barplot showing the number of orthogroups identified in one to the fifty-nine chlamydial genomes included in the analysis. A large majority of orthologous groups are restricted to one or only few genomes.

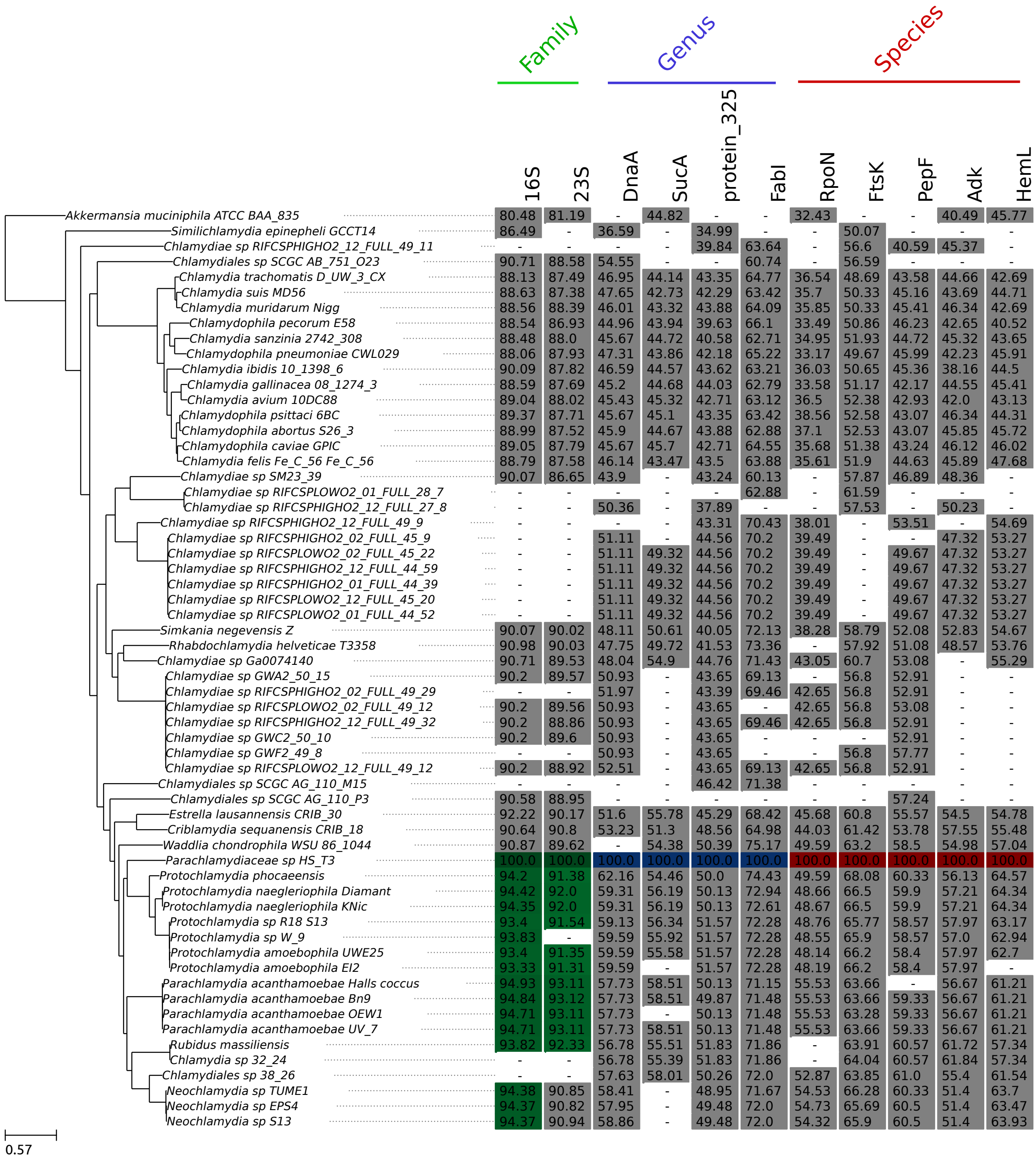

Taxon	16S	23S	DnaA	SucA	protein_325	FabI	RpoN	FtsK	PepF	Adk	HemL
Akkermansia muciniphila ATCC BAA_835	80.48	81.19	-	44.82	-	-	32.43	-	-	40.49	45.77
Similichlamydia epinepheli GCCT14	86.49	-	36.59	-	34.99	-	-	50.07	-	-	-
Chlamydiae sp RIFCSPHIGHO2_12_FULL_49_11	-	-	-	-	39.84	63.64	-	56.6	40.59	45.37	-
Chlamydiales sp SCGC AB_751_O23	90.71	88.58	54.55	-	-	60.74	-	56.59	-	-	-
Chlamydia trachomatis D_UW_3_CX	88.13	87.49	46.95	44.14	43.35	64.77	36.54	48.69	43.58	44.66	42.69
Chlamydia suis MD56	88.63	87.38	47.65	42.73	42.29	63.42	35.7	50.33	45.16	43.69	44.71
Chlamydia muridarum Nigg	88.56	88.39	46.01	43.32	43.88	64.09	35.85	50.33	45.41	46.34	42.69
Chlamydophila pecorum E58	88.54	86.93	44.96	43.94	39.63	66.1	33.49	50.86	46.23	42.65	40.52
Chlamydia sanzinia 2742_308	88.48	88.0	45.67	44.72	40.58	62.71	34.95	51.93	44.72	45.32	43.65
Chlamydophila pneumoniae CWL029	88.06	87.93	47.31	43.86	42.18	65.22	33.17	49.67	45.99	42.23	45.91
Chlamydia ibidis 10_1398_6	90.09	87.82	46.59	44.57	43.62	63.21	36.03	50.65	45.36	38.16	44.5
Chlamydia gallinacea 08_1274_3	88.59	87.69	45.2	44.68	44.03	62.79	33.58	51.17	42.17	44.55	45.41
Chlamydia avium 10DC88	89.04	88.02	45.43	45.32	42.71	63.12	36.5	52.38	42.93	42.0	43.13
Chlamydophila psittaci 6BC	89.37	87.71	45.67	45.1	43.35	63.42	38.56	52.58	43.07	46.34	44.31
Chlamydophila abortus S26_3	88.99	87.52	45.9	44.67	43.88	62.88	37.1	52.53	43.07	45.85	45.72
Chlamydophila caviae GPIC	89.05	87.79	45.67	45.7	42.71	64.55	35.68	51.38	43.24	46.12	46.02
Chlamydia felis Fe_C_56 Fe_C_56	88.79	87.58	46.14	43.47	43.5	63.88	35.61	51.9	44.63	45.89	47.68
Chlamydiae sp SM23_39	90.07	86.65	43.9	-	43.24	60.13	-	57.87	46.89	48.36	-
Chlamydiae sp RIFCSPLOWO2_01_FULL_28_7	-	-	-	-	-	62.88	-	61.59	-	-	-
Chlamydiae sp RIFCSPHIGHO2_12_FULL_27_8	-	-	50.36	-	37.89	-	-	57.53	-	50.23	-
Chlamydiae sp RIFCSPHIGHO2_12_FULL_49_9	-	-	-	-	43.31	70.43	38.01	-	53.51	-	54.69
Chlamydiae sp RIFCSPHIGHO2_02_FULL_45_9	-	-	51.11	-	44.56	70.2	39.49	-	-	47.32	53.27
Chlamydiae sp RIFCSPLOWO2_02_FULL_45_22	-	-	51.11	49.32	44.56	70.2	39.49	-	49.67	47.32	53.27
Chlamydiae sp RIFCSPHIGHO2_12_FULL_44_59	-	-	51.11	49.32	44.56	70.2	39.49	-	49.67	47.32	53.27
Chlamydiae sp RIFCSPHIGHO2_01_FULL_44_39	-	-	51.11	49.32	44.56	70.2	39.49	-	49.67	47.32	53.27
Chlamydiae sp RIFCSPLOWO2_12_FULL_45_20	-	-	51.11	49.32	44.56	70.2	39.49	-	49.67	47.32	53.27
Chlamydiae sp RIFCSPLOWO2_01_FULL_44_52	-	-	51.11	49.32	44.56	70.2	39.49	-	49.67	47.32	53.27
Simkania negevensis Z	90.07	90.02	48.11	50.61	40.05	72.13	38.28	58.79	52.08	52.83	54.67
Rhabdochlamydia helveticae T3358	90.98	90.03	47.75	49.72	41.53	73.36	-	57.92	51.08	48.57	53.76
Chlamydiae sp Ga0074140	90.71	89.53	48.04	54.9	44.76	71.43	43.05	60.7	53.08	-	55.29
Chlamydiae sp GWA2_50_15	90.2	89.57	50.93	-	43.65	69.13	-	56.8	52.91	-	-
Chlamydiae sp RIFCSPHIGHO2_02_FULL_49_29	-	-	51.97	-	43.39	69.46	42.65	56.8	52.91	-	-
Chlamydiae sp RIFCSPLOWO2_02_FULL_49_12	90.2	89.56	50.93	-	43.65	-	42.65	56.8	53.08	-	-
Chlamydiae sp RIFCSPHIGHO2_12_FULL_49_32	90.2	88.86	50.93	-	43.65	69.46	42.65	56.8	52.91	-	-
Chlamydiae sp GWC2_50_10	90.2	89.6	50.93	-	43.65	-	-	-	52.91	-	-
Chlamydiae sp GWF2_49_8	-	-	50.93	-	43.65	-	-	56.8	57.77	-	-
Chlamydiae sp RIFCSPLOWO2_12_FULL_49_12	90.2	88.92	52.51	-	43.65	69.13	42.65	56.8	52.91	-	-
Chlamydiales sp SCGC AG_110_M15	-	-	-	-	46.42	71.38	-	-	-	-	-
Chlamydiales sp SCGC AG_110_P3	90.58	88.95	-	-	-	-	-	-	57.24	-	-
Estrella lausannensis CRIB_30	92.22	90.17	51.6	55.78	45.29	68.42	45.68	60.8	55.57	54.5	54.78
Criblamydia sequanensis CRIB_18	90.64	90.8	53.23	51.3	48.56	64.98	44.03	61.42	53.78	57.55	55.48
Waddlia chondrophila WSU 86_1044	90.87	89.62	-	54.38	50.39	75.17	49.59	63.2	58.5	54.98	57.04
Parachlamydiaceae sp HS_T3	100.0	100.0	100.0	100.0	100.0	100.0	100.0	100.0	100.0	100.0	100.0
Protochlamydia phocaeensis	94.2	91.38	62.16	54.46	50.0	74.43	49.59	68.08	60.33	56.13	64.57
Protochlamydia naegleriophila Diamant	94.42	92.0	59.31	56.19	50.13	72.94	48.66	66.5	59.9	57.21	64.34
Protochlamydia naegleriophila KNic	94.35	92.0	59.31	56.19	50.13	72.61	48.67	66.5	59.9	57.21	64.34
Protochlamydia sp R18 S13	93.4	91.54	59.13	56.34	51.57	72.28	48.76	65.77	58.57	57.97	63.17
Protochlamydia sp W_9	93.83	-	59.59	55.92	51.57	72.28	48.55	65.9	58.57	57.0	62.94
Protochlamydia amoebophila UWE25	93.4	91.35	59.59	55.58	51.57	72.28	48.14	66.2	58.4	57.97	62.7
Protochlamydia amoebophila EI2	93.33	91.31	59.59	-	51.57	72.28	48.19	66.2	58.4	57.97	-
Parachlamydia acanthamoebae Halls coccus	94.93	93.11	57.73	58.51	50.13	71.15	55.53	63.66	-	56.67	61.21
Parachlamydia acanthamoebae Bn9	94.84	93.12	57.73	58.51	49.87	71.48	55.53	63.66	59.33	56.67	61.21
Parachlamydia acanthamoebae OEW1	94.71	93.11	57.73	-	50.13	71.48	55.53	63.28	59.33	56.67	61.21
Parachlamydia acanthamoebae UV_7	94.71	93.11	57.73	58.51	50.13	71.48	55.53	63.66	59.33	56.67	61.21
Rubidus massiliensis	93.82	92.33	56.78	55.51	51.83	71.86	-	63.91	60.57	61.72	57.34
Chlamydia sp 32_24	-	-	56.78	55.39	51.83	71.86	-	64.04	60.57	61.84	57.34
Chlamydiales sp 38_26	-	-	57.63	58.01	50.26	72.0	52.87	63.85	61.0	55.4	61.54
Neochlamydia sp TUME1	94.38	90.85	58.41	-	48.95	71.67	54.53	66.28	60.33	51.4	63.7
Neochlamydia sp EPS4	94.37	90.82	57.95	-	49.48	72.0	54.73	65.69	60.5	51.4	63.47
Neochlamydia sp S13	94.37	90.94	58.86	-	49.48	72.0	54.32	65.9	60.5	51.4	63.93

Figure S2: Percentage of nucleotide and amino acid identitiy of the nine taxonomic markers as compared to *Parachlamydiaceae* sp. HS-T3. Sequence conservation suggest that strains with **green rows** are part of the same family (*Parachlamydiaceae*). The low conservation of DnaA, SucA, protein 325 and FabI supports classification of *Parachlamydiaceae* sp HS_T3 as the unique representative of a new *Parachlamydiaceae* genus.

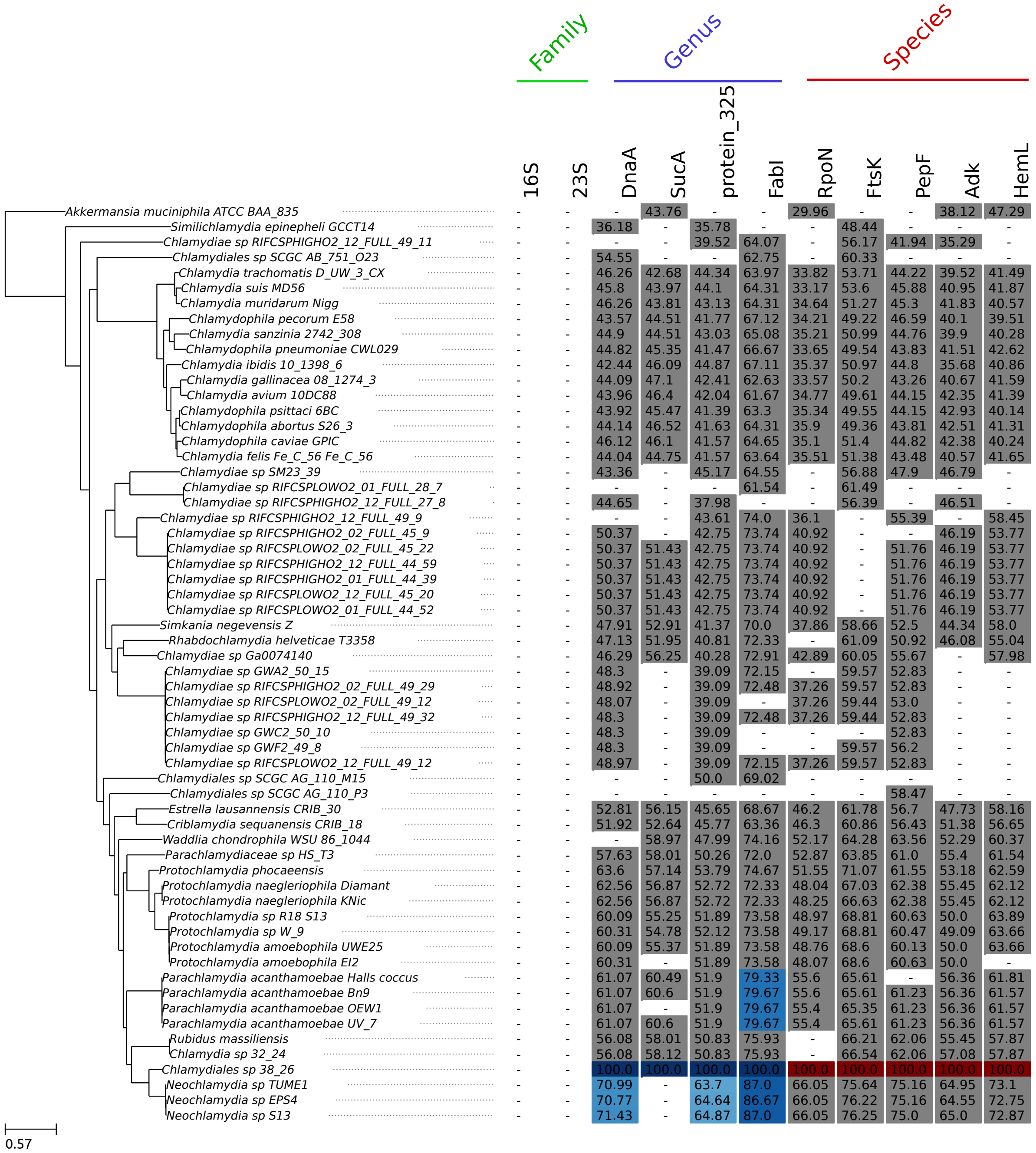

Taxon	16S	23S	DnaA	SucA	protein_325	FabI	RpoN	FtsK	PepF	Adk	HemL
Akkermansia muciniphila ATCC BAA_835	-	-	-	43.76	-	-	29.96	-	-	38.12	47.29
Similichlamydia epinepheli GCCT14	-	-	36.18	-	35.78	-	-	48.44	-	-	-
Chlamydiae sp RIFCSPHIGHO2_12_FULL_49_11	-	-	-	-	39.52	64.07	-	56.17	41.94	35.29	-
Chlamydiales sp SCGC AB_751_O23	-	-	54.55	-	-	62.75	-	60.33	-	-	-
Chlamydia trachomatis D_UW_3_CX	-	-	46.26	42.68	44.34	63.97	33.82	53.71	44.22	39.52	41.49
Chlamydia suis MD56	-	-	45.8	43.97	44.1	64.31	33.17	53.6	45.88	40.95	41.87
Chlamydia muridarum Nigg	-	-	46.26	43.81	43.13	64.31	34.64	51.27	45.3	41.83	40.57
Chlamydophila pecorum E58	-	-	43.57	44.51	41.77	67.12	34.21	49.22	46.59	40.1	39.51
Chlamydia sanzinia 2742_308	-	-	44.9	44.51	43.03	65.08	35.21	50.99	44.76	39.9	40.28
Chlamydophila pneumoniae CWL029	-	-	44.82	45.35	41.47	66.67	33.65	49.54	43.83	41.51	42.62
Chlamydia ibidis 10_1398_6	-	-	42.44	46.09	44.87	67.11	35.37	50.97	44.8	35.68	40.86
Chlamydia gallinacea 08_1274_3	-	-	44.09	47.1	42.41	62.63	33.57	50.2	43.26	40.67	41.59
Chlamydia avium 10DC88	-	-	43.96	46.4	42.04	61.67	34.77	49.61	44.15	42.35	41.39
Chlamydophila psittaci 6BC	-	-	43.92	45.47	41.39	63.3	35.34	49.55	44.15	42.93	40.14
Chlamydophila abortus S26_3	-	-	44.14	46.52	41.63	64.31	35.9	49.36	43.81	42.51	41.31
Chlamydophila caviae GPIC	-	-	46.12	46.1	41.57	64.65	35.1	51.4	44.82	42.38	40.24
Chlamydia felis Fe_C_56 Fe_C_56	-	-	44.04	44.75	41.57	63.64	35.51	51.38	43.48	40.57	41.65
Chlamydiae sp SM23_39	-	-	43.36	-	45.17	64.55	-	56.88	47.9	46.79	-
Chlamydiae sp RIFCSPLOWO2_01_FULL_28_7	-	-	-	-	-	61.54	-	61.49	-	-	-
Chlamydiae sp RIFCSPHIGHO2_12_FULL_27_8	-	-	44.65	-	37.98	-	-	56.39	-	46.51	-
Chlamydiae sp RIFCSPHIGHO2_12_FULL_49_9	-	-	-	-	43.61	74.0	36.1	-	55.39	-	58.45
Chlamydiae sp RIFCSPHIGHO2_02_FULL_45_9	-	-	50.37	-	42.75	73.74	40.92	-	-	46.19	53.77
Chlamydiae sp RIFCSPLOWO2_02_FULL_45_22	-	-	50.37	51.43	42.75	73.74	40.92	-	51.76	46.19	53.77
Chlamydiae sp RIFCSPHIGHO2_12_FULL_44_59	-	-	50.37	51.43	42.75	73.74	40.92	-	51.76	46.19	53.77
Chlamydiae sp RIFCSPHIGHO2_01_FULL_44_39	-	-	50.37	51.43	42.75	73.74	40.92	-	51.76	46.19	53.77
Chlamydiae sp RIFCSPLOWO2_12_FULL_45_20	-	-	50.37	51.43	42.75	73.74	40.92	-	51.76	46.19	53.77
Chlamydiae sp RIFCSPLOWO2_01_FULL_44_52	-	-	50.37	51.43	42.75	73.74	40.92	-	51.76	46.19	53.77
Simkania negevensis Z	-	-	47.91	52.91	41.37	70.0	37.86	58.66	52.5	44.34	58.0
Rhabdochlamydia helveticae T3358	-	-	47.13	51.95	40.81	72.33	-	61.09	50.92	46.08	55.04
Chlamydiae sp Ga0074140	-	-	46.29	56.25	40.28	72.91	42.89	60.05	55.67	-	57.98
Chlamydiae sp GWA2_50_15	-	-	48.3	-	39.09	72.15	-	59.57	52.83	-	-
Chlamydiae sp RIFCSPHIGHO2_02_FULL_49_29	-	-	48.92	-	39.09	72.48	37.26	59.57	52.83	-	-
Chlamydiae sp RIFCSPLOWO2_02_FULL_49_12	-	-	48.07	-	39.09	-	37.26	59.44	53.0	-	-
Chlamydiae sp RIFCSPHIGHO2_12_FULL_49_32	-	-	48.3	-	39.09	72.48	37.26	59.44	52.83	-	-
Chlamydiae sp GWC2_50_10	-	-	48.3	-	39.09	-	-	-	52.83	-	-
Chlamydiae sp GWF2_49_8	-	-	48.3	-	39.09	-	-	59.57	56.2	-	-
Chlamydiae sp RIFCSPLOWO2_12_FULL_49_12	-	-	48.97	-	39.09	72.15	37.26	59.57	52.83	-	-
Chlamydiales sp SCGC AG_110_M15	-	-	-	-	50.0	69.02	-	-	-	-	-
Chlamydiales sp SCGC AG_110_P3	-	-	-	-	-	-	-	-	58.47	-	-
Estrella lausannensis CRIB_30	-	-	52.81	56.15	45.65	68.67	46.2	61.78	56.7	47.73	58.16
Criblamydia sequanensis CRIB_18	-	-	51.92	52.64	45.77	63.36	46.3	60.86	56.43	51.38	56.65
Waddlia chondrophila WSU 86_1044	-	-	-	58.97	47.99	74.16	52.17	64.28	63.56	52.29	60.37
Parachlamydiaceae sp HS_T3	-	-	57.63	58.01	50.26	72.0	52.87	63.85	61.0	55.4	61.54
Protochlamydia phocaeensis	-	-	63.6	57.14	53.79	74.67	51.55	71.07	61.55	53.18	62.59
Protochlamydia naegleriophila Diamant	-	-	62.56	56.87	52.72	72.33	48.04	67.03	62.38	55.45	62.12
Protochlamydia naegleriophila KNic	-	-	62.56	56.87	52.72	72.33	48.25	66.63	62.38	55.45	62.12
Protochlamydia sp R18 S13	-	-	60.09	55.25	51.89	73.58	48.97	68.81	60.63	50.0	63.89
Protochlamydia sp W_9	-	-	60.31	54.78	52.12	73.58	49.17	68.81	60.47	49.09	63.66
Protochlamydia amoebophila UWE25	-	-	60.09	55.37	51.89	73.58	48.76	68.6	60.13	50.0	63.66
Protochlamydia amoebophila EI2	-	-	60.31	-	51.89	73.58	48.07	68.6	60.63	50.0	-
Parachlamydia acanthamoebae Halls coccus	-	-	61.07	60.49	51.9	79.33	55.6	65.61	-	56.36	61.81
Parachlamydia acanthamoebae Bn9	-	-	61.07	60.6	51.9	79.67	55.6	65.61	61.23	56.36	61.57
Parachlamydia acanthamoebae OEW1	-	-	61.07	-	51.9	79.67	55.4	65.35	61.23	56.36	61.57
Parachlamydia acanthamoebae UV_7	-	-	61.07	60.6	51.9	79.67	55.4	65.61	61.23	56.36	61.57
Rubidus massiliensis	-	-	56.08	58.01	50.83	75.93	-	66.21	62.06	55.45	57.87
Chlamydia sp 32_24	-	-	56.08	58.12	50.83	75.93	-	66.54	62.06	57.08	57.87
Chlamydiales sp 38_26	-	-	100.0	100.0	100.0	100.0	100.0	100.0	100.0	100.0	100.0
Neochlamydia sp TUME1	-	-	70.99	-	63.7	87.0	66.05	75.64	75.16	64.95	73.1
Neochlamydia sp EPS4	-	-	70.77	-	64.64	86.67	66.05	76.22	75.16	64.55	72.75
Neochlamydia sp S13	-	-	71.43	-	64.87	87.0	66.05	76.25	75.0	65.0	72.87

Figure S3: Percentage of nucleotide and amino acid identity of the nine taxonomic markers as compared to *Chlamydiales* sp. 38_26. The 16S and 23S rRNA sequences were missing from the metagenomic bin, but the relatively high conservation of DnaA, SucA, protein 325 and FabI and low conservation of species-level markers (RpoN, FtsK, PepF, Adk and HemL) suggest that the *Chlamydiales* sp. 38_26 is a new species of the genus *Neochlamydia*.

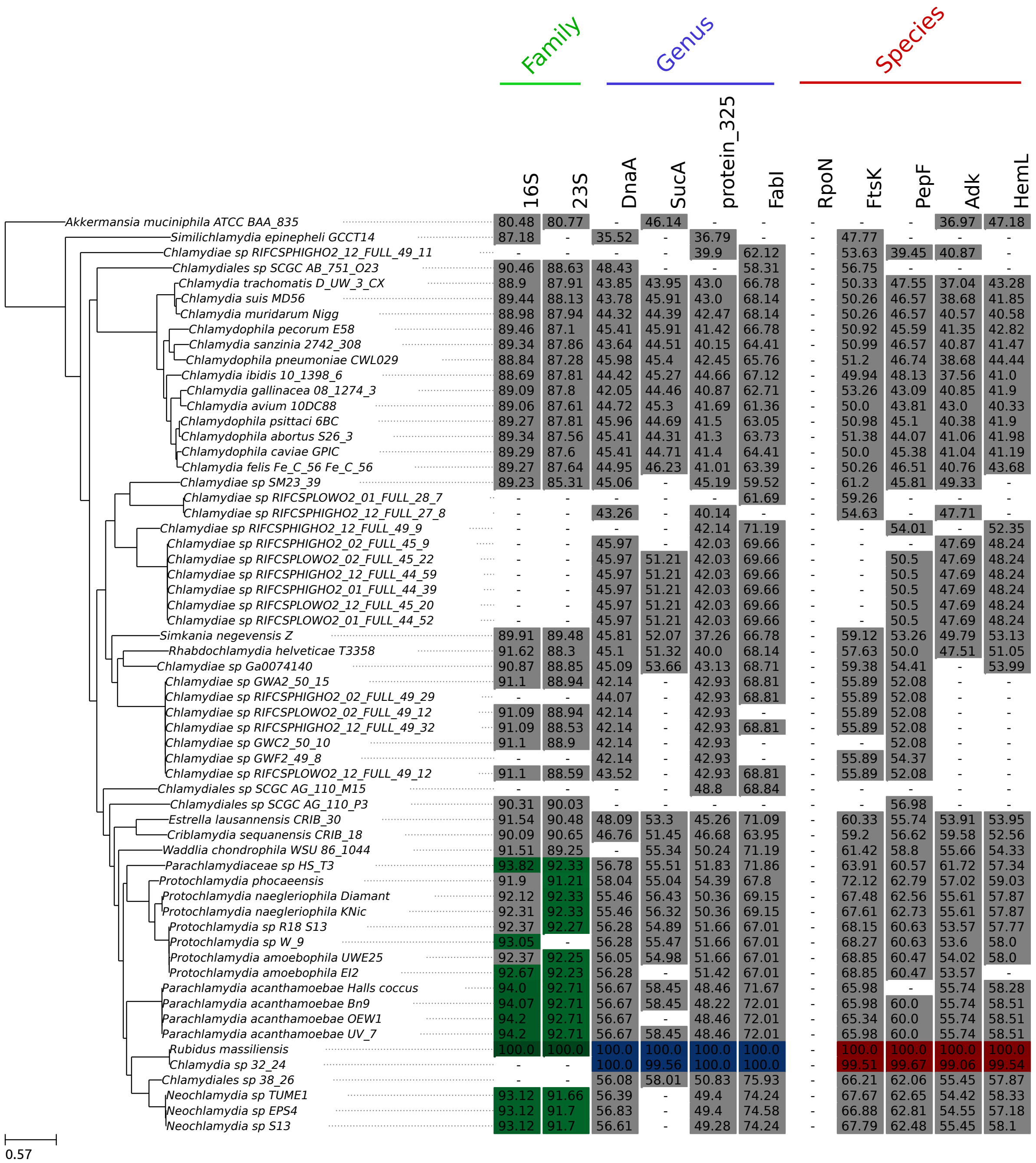

Figure S4: Percentage of nucleotide and amino acid identity of the nine taxonomic markers as compared to *Rubidus massiliensis*. Sequence conservation of both genus and species-level taxonomic markers indicate that *Chlamydia* sp. 32_24 is a new *Rubidus massiliensis* strain.

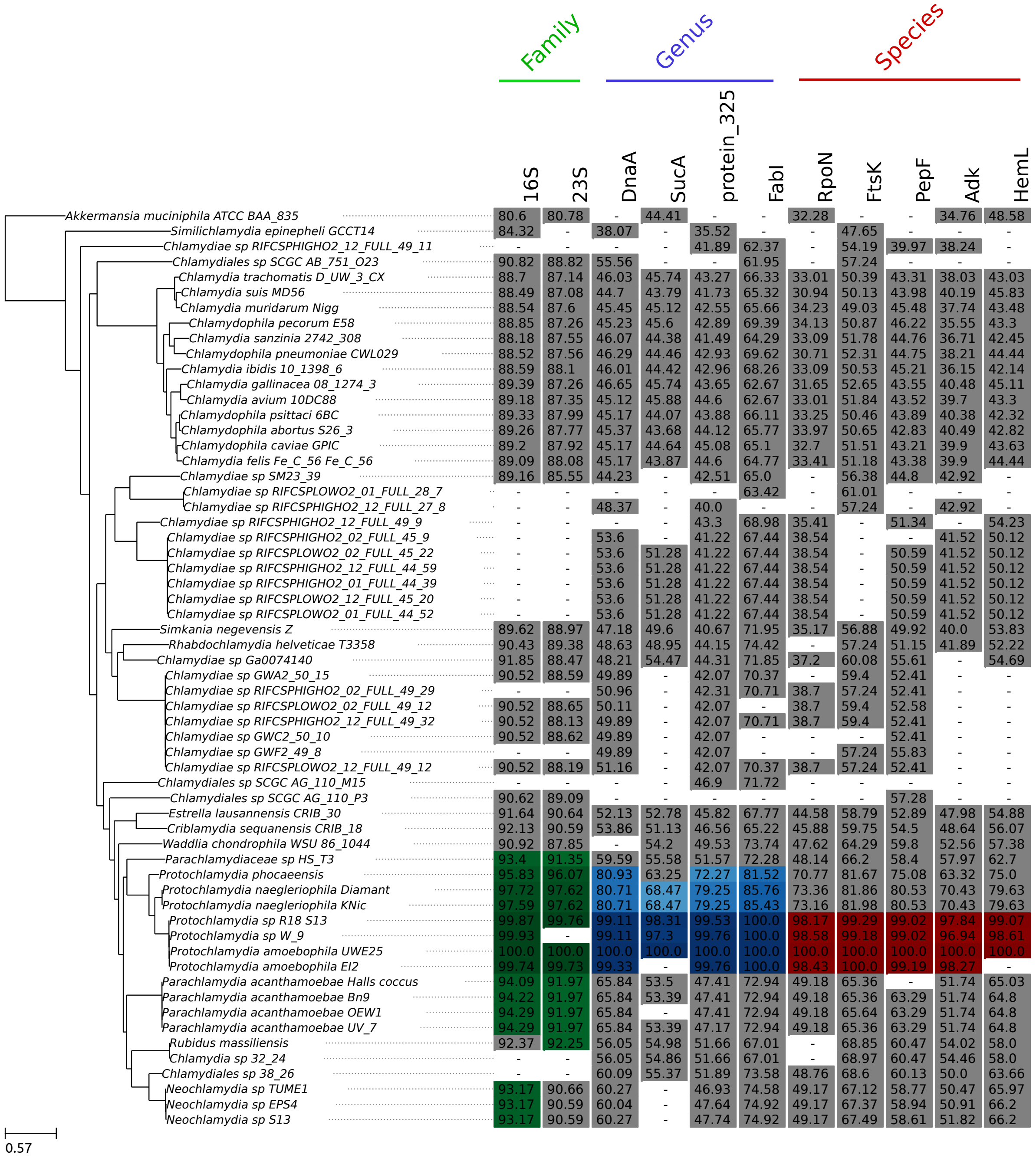

Taxon	16S	23S	DnaA	SucA	protein_325	FabI	RpoN	FtsK	PepF	Adk	HemL
Akkermansia muciniphila ATCC BAA_835	80.6	80.78	-	44.41	-	-	32.28	-	-	34.76	48.58
Similichlamydia epinepheli GCCT14	84.32	-	38.07	-	35.52	-	-	47.65	-	-	-
Chlamydiae sp RIFCSPHIGHO2_12_FULL_49_11	-	-	-	-	41.89	62.37	-	54.19	39.97	38.24	-
Chlamydiales sp SCGC AB_751_O23	90.82	88.82	55.56	-	-	61.95	-	57.24	-	-	-
Chlamydia trachomatis D_UW_3_CX	88.7	87.14	46.03	45.74	43.27	66.33	33.01	50.39	43.31	38.03	43.03
Chlamydia suis MD56	88.49	87.08	44.7	43.79	41.73	65.32	30.94	50.13	43.98	40.19	45.83
Chlamydia muridarum Nigg	88.54	87.6	45.45	45.12	42.55	65.66	34.23	49.03	45.48	37.74	43.48
Chlamydophila pecorum E58	88.85	87.26	45.23	45.6	42.89	69.39	34.13	50.87	46.22	35.55	43.3
Chlamydia sanzinia 2742_308	88.18	87.55	46.07	44.38	41.49	64.29	33.09	51.78	44.76	36.71	42.45
Chlamydophila pneumoniae CWL029	88.52	87.56	46.29	44.46	42.93	69.62	30.71	52.31	44.75	38.21	44.44
Chlamydia ibidis 10_1398_6	88.59	88.1	46.01	44.42	42.96	68.26	33.09	50.53	45.21	36.15	42.14
Chlamydia gallinacea 08_1274_3	89.39	87.26	46.65	45.74	43.65	62.67	31.65	52.65	43.55	40.48	45.11
Chlamydia avium 10DC88	89.18	87.35	45.12	45.88	44.6	62.67	33.01	51.84	43.52	39.7	43.3
Chlamydophila psittaci 6BC	89.33	87.99	45.17	44.07	43.88	66.11	33.25	50.46	43.89	40.38	42.32
Chlamydophila abortus S26_3	89.26	87.77	45.37	43.68	44.12	65.77	33.97	50.65	42.83	40.49	42.82
Chlamydophila caviae GPIC	89.2	87.92	45.17	44.64	45.08	65.1	32.7	51.51	43.21	39.9	43.63
Chlamydia felis Fe_C_56 Fe_C_56	89.09	88.08	45.17	43.87	44.6	64.77	33.41	51.18	43.38	39.9	44.44
Chlamydiae sp SM23_39	89.16	85.55	44.23	-	42.51	65.0	-	56.38	44.8	42.92	-
Chlamydiae sp RIFCSPLOWO2_01_FULL_28_7	-	-	-	-	-	63.42	-	61.01	-	-	-
Chlamydiae sp RIFCSPHIGHO2_12_FULL_27_8	-	-	48.37	-	40.0	-	-	57.24	-	42.92	-
Chlamydiae sp RIFCSPHIGHO2_12_FULL_49_9	-	-	-	-	43.3	68.98	35.41	-	51.34	-	54.23
Chlamydiae sp RIFCSPHIGHO2_02_FULL_45_9	-	-	53.6	-	41.22	67.44	38.54	-	-	41.52	50.12
Chlamydiae sp RIFCSPLOWO2_02_FULL_45_22	-	-	53.6	51.28	41.22	67.44	38.54	-	50.59	41.52	50.12
Chlamydiae sp RIFCSPHIGHO2_12_FULL_44_59	-	-	53.6	51.28	41.22	67.44	38.54	-	50.59	41.52	50.12
Chlamydiae sp RIFCSPHIGHO2_01_FULL_44_39	-	-	53.6	51.28	41.22	67.44	38.54	-	50.59	41.52	50.12
Chlamydiae sp RIFCSPLOWO2_12_FULL_45_20	-	-	53.6	51.28	41.22	67.44	38.54	-	50.59	41.52	50.12
Chlamydiae sp RIFCSPLOWO2_01_FULL_44_52	-	-	53.6	51.28	41.22	67.44	38.54	-	50.59	41.52	50.12
Simkania negevensis Z	89.62	88.97	47.18	49.6	40.67	71.95	35.17	56.88	49.92	40.0	53.83
Rhabdochlamydia helveticae T3358	90.43	89.38	48.63	48.95	44.15	74.42	-	57.24	51.15	41.89	52.22
Chlamydiae sp Ga0074140	91.85	88.47	48.21	54.47	44.31	71.85	37.2	60.08	55.61	-	54.69
Chlamydiae sp GWA2_50_15	90.52	88.59	49.89	-	42.07	70.37	-	59.4	52.41	-	-
Chlamydiae sp RIFCSPHIGHO2_02_FULL_49_29	-	-	50.96	-	42.31	70.71	38.7	57.24	52.41	-	-
Chlamydiae sp RIFCSPLOWO2_02_FULL_49_12	90.52	88.65	50.11	-	42.07	-	38.7	59.4	52.58	-	-
Chlamydiae sp RIFCSPHIGHO2_12_FULL_49_32	90.52	88.13	49.89	-	42.07	70.71	38.7	59.4	52.41	-	-
Chlamydiae sp GWC2_50_10	90.52	88.62	49.89	-	42.07	-	-	-	52.41	-	-
Chlamydiae sp GWF2_49_8	-	-	49.89	-	42.07	-	-	57.24	55.83	-	-
Chlamydiae sp RIFCSPLOWO2_12_FULL_49_12	90.52	88.19	51.16	-	42.07	70.37	38.7	57.24	52.41	-	-
Chlamydiales sp SCGC AG_110_M15	-	-	-	-	46.9	71.72	-	-	-	-	-
Chlamydiales sp SCGC AG_110_P3	90.62	89.09	-	-	-	-	-	-	57.28	-	-
Estrella lausannensis CRIB_30	91.64	90.64	52.13	52.78	45.82	67.77	44.58	58.79	52.89	47.98	54.88
Criblamydia sequanensis CRIB_18	92.13	90.59	53.86	51.13	46.56	65.22	45.88	59.75	54.5	48.64	56.07
Waddlia chondrophila WSU 86_1044	90.92	87.85	-	54.2	49.53	73.74	47.62	64.29	59.8	52.56	57.38
Parachlamydiaceae sp HS_T3	93.4	91.35	59.59	55.58	51.57	72.28	48.14	66.2	58.4	57.97	62.7
Protochlamydia phocaeensis	95.83	96.07	80.93	63.25	72.27	81.52	70.77	81.67	75.08	63.32	75.0
Protochlamydia naegleriophila Diamant	97.72	97.62	80.71	68.47	79.25	85.76	73.36	81.86	80.53	70.43	79.63
Protochlamydia naegleriophila KNic	97.59	97.62	80.71	68.47	79.25	85.43	73.16	81.98	80.53	70.43	79.63
Protochlamydia sp R18 S13	99.87	99.76	99.11	98.31	99.53	100.0	98.17	99.29	99.02	97.84	99.07
Protochlamydia sp W_9	99.93	-	99.11	97.3	99.76	100.0	98.58	99.18	99.02	96.94	98.61
Protochlamydia amoebophila UWE25	100.0	100.0	100.0	100.0	100.0	100.0	100.0	100.0	100.0	100.0	100.0
Protochlamydia amoebophila EI2	99.74	99.73	99.33	-	99.76	100.0	98.43	100.0	99.19	98.27	-
Parachlamydia acanthamoebae Halls coccus	94.09	91.97	65.84	53.5	47.41	72.94	49.18	65.36	-	51.74	65.03
Parachlamydia acanthamoebae Bn9	94.22	91.97	65.84	53.39	47.41	72.94	49.18	65.36	63.29	51.74	64.8
Parachlamydia acanthamoebae OEW1	94.29	91.97	65.84	-	47.41	72.94	49.18	65.64	63.29	51.74	64.8
Parachlamydia acanthamoebae UV_7	94.29	91.97	65.84	53.39	47.17	72.94	49.18	65.36	63.29	51.74	64.8
Rubidus massiliensis	92.37	92.25	56.05	54.98	51.66	67.01	-	68.85	60.47	54.02	58.0
Chlamydia sp 32_24	-	-	56.05	54.86	51.66	67.01	-	68.97	60.47	54.46	58.0
Chlamydiales sp 38_26	-	-	60.09	55.37	51.89	73.58	48.76	68.6	60.13	50.0	63.66
Neochlamydia sp TUME1	93.17	90.66	60.27	-	46.93	74.58	49.17	67.12	58.77	50.47	65.97
Neochlamydia sp EPS4	93.17	90.59	60.04	-	47.64	74.92	49.17	67.37	58.94	50.91	66.2
Neochlamydia sp S13	93.17	90.59	60.27	-	47.74	74.92	49.17	67.49	58.61	51.82	66.2

Figure S5: Percentage of nucleotide and amino acid identity of the nine taxonomic markers as compared to *Protochlamydia amoebophila*. Sequence conservation of DnaA, SucA, protein 325 and FabI indicates that *Protochlamydia* sp. R18 S13 and *Protochlamydia* sp. W_9 are *P. amoebophila* strains.

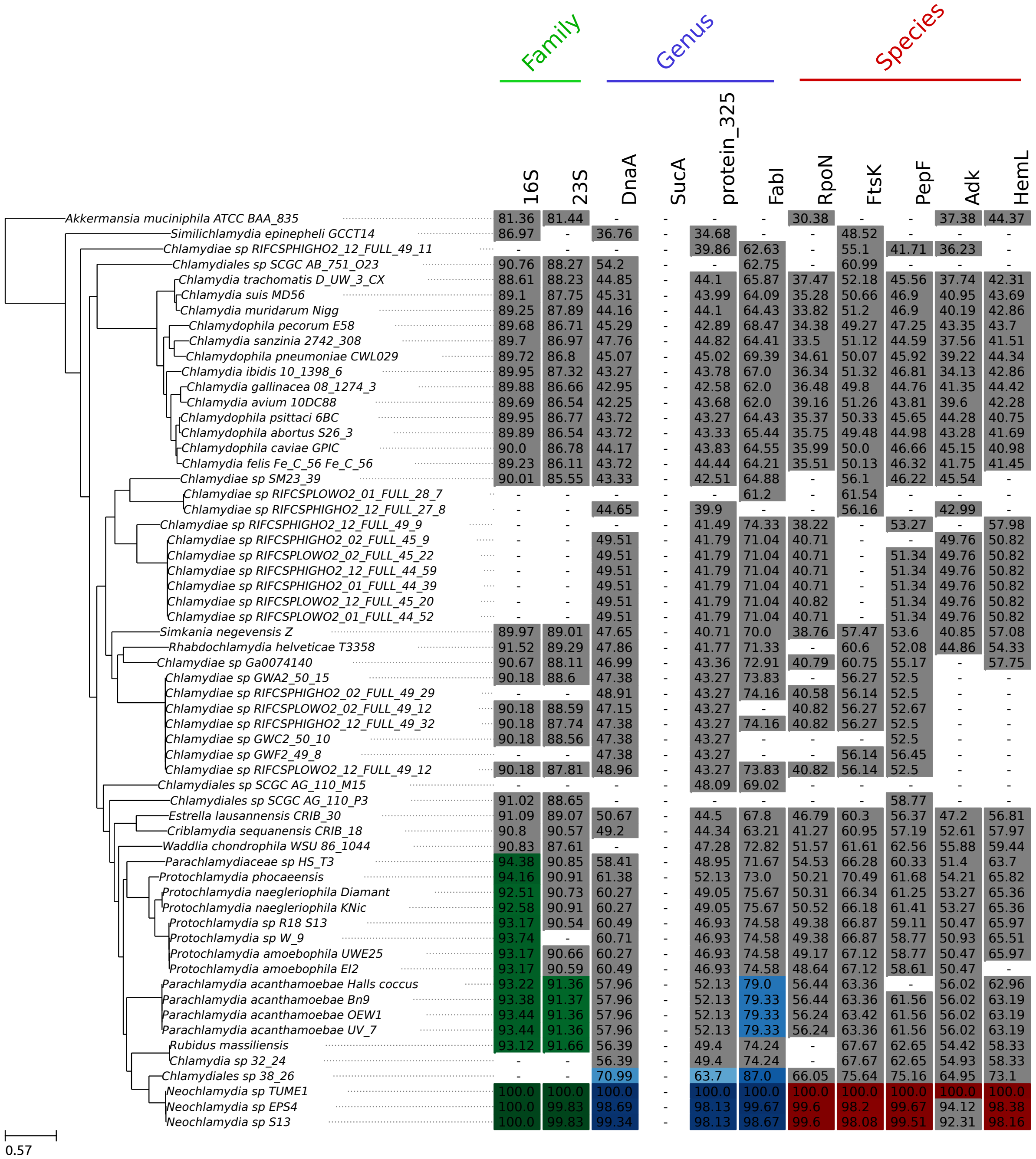

	16S	23S	DnaA	SucA	protein_325	FabI	RpoN	FtsK	PepF	Adk	HemL
Akkermansia muciniphila ATCC BAA_835	81.36	81.44	-	-	-	-	30.38	-	-	37.38	44.37
Similichlamydia epinepheli GCCT14	86.97	-	36.76	-	34.68	-	-	48.52	-	-	-
Chlamydiae sp RIFCSPHIGHO2_12_FULL_49_11	-	-	-	-	39.86	62.63	-	55.1	41.71	36.23	-
Chlamydiales sp SCGC AB_751_O23	90.76	88.27	54.2	-	-	62.75	-	60.99	-	-	-
Chlamydia trachomatis D_UW_3_CX	88.61	88.23	44.85	-	44.1	65.87	37.47	52.18	45.56	37.74	42.31
Chlamydia suis MD56	89.1	87.75	45.31	-	43.99	64.09	35.28	50.66	46.9	40.95	43.69
Chlamydia muridarum Nigg	89.25	87.89	44.16	-	44.1	64.43	33.82	51.2	46.9	40.19	42.86
Chlamydophila pecorum E58	89.68	86.71	45.29	-	42.89	68.47	34.38	49.27	47.25	43.35	43.7
Chlamydia sanzinia 2742_308	89.7	86.97	47.76	-	44.82	64.41	33.5	51.12	44.59	37.56	41.51
Chlamydophila pneumoniae CWL029	89.72	86.8	45.07	-	45.02	69.39	34.61	50.07	45.92	39.22	44.34
Chlamydia ibidis 10_1398_6	89.95	87.32	43.27	-	43.78	67.0	36.34	51.32	46.81	34.13	42.86
Chlamydia gallinacea 08_1274_3	89.88	86.66	42.95	-	42.58	62.0	36.48	49.8	44.76	41.35	44.42
Chlamydia avium 10DC88	89.69	86.54	42.25	-	43.68	62.0	39.16	51.26	43.81	39.6	42.28
Chlamydophila psittaci 6BC	89.95	86.77	43.72	-	43.27	64.43	35.37	50.33	45.65	44.28	40.75
Chlamydophila abortus S26_3	89.89	86.54	43.72	-	43.33	65.44	35.75	49.48	44.98	43.28	41.69
Chlamydophila caviae GPIC	90.0	86.78	44.17	-	43.83	64.55	35.99	50.0	46.66	45.15	40.98
Chlamydia felis Fe_C_56 Fe_C_56	89.23	86.11	43.72	-	44.44	64.21	35.51	50.13	46.32	41.75	41.45
Chlamydiae sp SM23_39	90.01	85.55	43.33	-	42.51	64.88	-	56.1	46.22	45.54	-
Chlamydiae sp RIFCSPLOWO2_01_FULL_28_7	-	-	-	-	-	61.2	-	61.54	-	-	-
Chlamydiae sp RIFCSPHIGHO2_12_FULL_27_8	-	-	44.65	-	39.9	-	-	56.16	-	42.99	-
Chlamydiae sp RIFCSPHIGHO2_12_FULL_49_9	-	-	-	-	41.49	74.33	38.22	-	53.27	-	57.98
Chlamydiae sp RIFCSPHIGHO2_02_FULL_45_9	-	-	49.51	-	41.79	71.04	40.71	-	-	49.76	50.82
Chlamydiae sp RIFCSPLOWO2_02_FULL_45_22	-	-	49.51	-	41.79	71.04	40.71	-	51.34	49.76	50.82
Chlamydiae sp RIFCSPHIGHO2_12_FULL_44_59	-	-	49.51	-	41.79	71.04	40.71	-	51.34	49.76	50.82
Chlamydiae sp RIFCSPHIGHO2_01_FULL_44_39	-	-	49.51	-	41.79	71.04	40.71	-	51.34	49.76	50.82
Chlamydiae sp RIFCSPLOWO2_12_FULL_45_20	-	-	49.51	-	41.79	71.04	40.82	-	51.34	49.76	50.82
Chlamydiae sp RIFCSPLOWO2_01_FULL_44_52	-	-	49.51	-	41.79	71.04	40.71	-	51.34	49.76	50.82
Simkania negevensis Z	89.97	89.01	47.65	-	40.71	70.0	38.76	57.47	53.6	40.85	57.08
Rhabdochlamydia helveticae T3358	91.52	89.29	47.86	-	41.77	71.33	-	60.6	52.08	44.86	54.33
Chlamydiae sp Ga0074140	90.67	88.11	46.99	-	43.36	72.91	40.79	60.75	55.17	-	57.75
Chlamydiae sp GWA2_50_15	90.18	88.6	47.38	-	43.27	73.83	-	56.27	52.5	-	-
Chlamydiae sp RIFCSPHIGHO2_02_FULL_49_29	-	-	48.91	-	43.27	74.16	40.58	56.14	52.5	-	-
Chlamydiae sp RIFCSPLOWO2_02_FULL_49_12	90.18	88.59	47.15	-	43.27	-	40.82	56.27	52.67	-	-
Chlamydiae sp RIFCSPHIGHO2_12_FULL_49_32	90.18	87.74	47.38	-	43.27	74.16	40.82	56.27	52.5	-	-
Chlamydiae sp GWC2_50_10	90.18	88.56	47.38	-	43.27	-	-	-	52.5	-	-
Chlamydiae sp GWF2_49_8	-	-	47.38	-	43.27	-	-	56.14	56.45	-	-
Chlamydiae sp RIFCSPLOWO2_12_FULL_49_12	90.18	87.81	48.96	-	43.27	73.83	40.82	56.14	52.5	-	-
Chlamydiales sp SCGC AG_110_M15	-	-	-	-	48.09	69.02	-	-	-	-	-
Chlamydiales sp SCGC AG_110_P3	91.02	88.65	-	-	-	-	-	-	58.77	-	-
Estrella lausannensis CRIB_30	91.09	89.07	50.67	-	44.5	67.8	46.79	60.3	56.37	47.2	56.81
Criblamydia sequanensis CRIB_18	90.8	90.57	49.2	-	44.34	63.21	41.27	60.95	57.19	52.61	57.97
Waddlia chondrophila WSU 86_1044	90.83	87.61	-	-	47.28	72.82	51.57	61.61	62.56	55.88	59.44
Parachlamydiaceae sp HS_T3	94.38	90.85	58.41	-	48.95	71.67	54.53	66.28	60.33	51.4	63.7
Protochlamydia phocaeensis	94.16	90.91	61.38	-	52.13	73.0	50.21	70.49	61.68	54.21	65.82
Protochlamydia naegleriophila Diamant	92.51	90.73	60.27	-	49.05	75.67	50.31	66.34	61.25	53.27	65.36
Protochlamydia naegleriophila KNic	92.58	90.91	60.27	-	49.05	75.67	50.52	66.18	61.41	53.27	65.36
Protochlamydia sp R18 S13	93.17	90.54	60.49	-	46.93	74.58	49.38	66.87	59.11	50.47	65.97
Protochlamydia sp W_9	93.74	-	60.71	-	46.93	74.58	49.38	66.87	58.77	50.93	65.51
Protochlamydia amoebophila UWE25	93.17	90.66	60.27	-	46.93	74.58	49.17	67.12	58.77	50.47	65.97
Protochlamydia amoebophila EI2	93.17	90.59	60.49	-	46.93	74.58	48.64	67.12	58.61	50.47	-
Parachlamydia acanthamoebae Halls coccus	93.22	91.36	57.96	-	52.13	79.0	56.44	63.36	-	56.02	62.96
Parachlamydia acanthamoebae Bn9	93.38	91.37	57.96	-	52.13	79.33	56.44	63.36	61.56	56.02	63.19
Parachlamydia acanthamoebae OEW1	93.44	91.36	57.96	-	52.13	79.33	56.24	63.42	61.56	56.02	63.19
Parachlamydia acanthamoebae UV_7	93.44	91.36	57.96	-	52.13	79.33	56.24	63.36	61.56	56.02	63.19
Rubidus massiliensis	93.12	91.66	56.39	-	49.4	74.24	-	67.67	62.65	54.42	58.33
Chlamydia sp 32_24	-	-	56.39	-	49.4	74.24	-	67.67	62.65	54.93	58.33
Chlamydiales sp 38_26	-	-	70.99	-	63.7	87.0	66.05	75.64	75.16	64.95	73.1
Neochlamydia sp TUME1	100.0	100.0	100.0	-	100.0	100.0	100.0	100.0	100.0	100.0	100.0
Neochlamydia sp EPS4	100.0	99.83	98.69	-	98.13	99.67	99.6	98.2	99.67	94.12	98.38
Neochlamydia sp S13	100.0	99.83	99.34	-	98.13	98.67	99.6	98.08	99.51	92.31	98.16

Figure S6: Percentage of nucleotide and amino acid identity of the nine taxonomic markers as compared to Neochlamydia sp. 1. Sequence conservation of both genus and species-level taxonomic markers indicates that the three *Neochlamydia* assemblies are representatives of the same species.

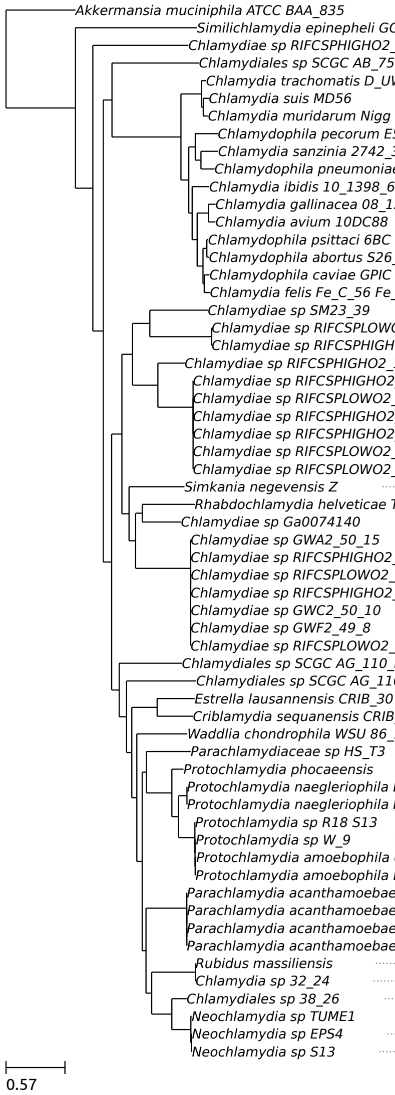

Taxon	16S	23S	DnaA	SucA	protein_325	FabI	RpoN	FtsK	PepF	Adk	HemL
	Family		Genus				Species				
Akkermansia muciniphila ATCC BAA_835	-	-	-	43.5	-	-	33.85	-	-	34.01	48.11
Similichlamydia epinepheli GCCT14	-	-	37.77	-	35.44	-	-	-	-	-	-
Chlamydiae sp RIFCSPHIGHO2_12_FULL_49_11	-	-	-	-	40.58	61.49	-	-	39.79	41.62	-
Chlamydiales sp SCGC AB_751_O23	-	-	47.29	-	-	58.64	-	-	-	-	-
Chlamydia trachomatis D_UW_3_CX	-	-	44.25	43.06	40.88	62.37	34.05	-	44.11	38.57	42.75
Chlamydia suis MD56	-	-	45.45	42.49	38.5	64.75	33.7	-	43.4	39.34	41.02
Chlamydia muridarum Nigg	-	-	44.47	42.96	42.48	63.73	34.15	-	43.56	40.48	42.17
Chlamydophila pecorum E58	-	-	44.25	43.82	39.28	65.76	38.5	-	42.74	40.29	43.07
Chlamydia sanzinia 2742_308	-	-	44.01	45.03	38.8	62.5	34.77	-	41.79	37.86	44.55
Chlamydophila pneumoniae CWL029	-	-	47.43	44.93	41.55	65.98	35.58	-	42.83	38.42	44.02
Chlamydia ibidis 10_1398_6	-	-	42.3	44.65	41.61	65.2	30.45	-	43.14	40.1	43.68
Chlamydia gallinacea 08_1274_3	-	-	42.72	44.28	41.65	60.74	32.89	-	42.5	40.29	47.14
Chlamydia avium 10DC88	-	-	43.35	45.83	41.12	61.74	34.15	-	42.36	43.41	44.84
Chlamydophila psittaci 6BC	-	-	43.52	44.73	42.17	62.16	31.69	-	44.24	42.79	44.44
Chlamydophila abortus S26_3	-	-	43.28	44.11	42.72	62.16	32.71	-	42.9	43.28	45.19
Chlamydophila caviae GPIC	-	-	43.52	44.43	40.78	63.51	34.22	-	43.22	46.04	41.78
Chlamydia felis Fe_C_56 Fe_C_56	-	-	42.79	45.02	41.26	63.85	31.51	-	43.51	41.79	42.89
Chlamydiae sp SM23_39	-	-	46.62	-	46.84	63.76	-	-	46.75	42.59	-
Chlamydiae sp RIFCSPLOWO2_01_FULL_28_7	-	-	-	-	-	63.51	-	-	-	-	-
Chlamydiae sp RIFCSPHIGHO2_12_FULL_27_8	-	-	46.62	-	38.69	-	-	-	-	43.19	-
Chlamydiae sp RIFCSPHIGHO2_12_FULL_49_9	-	-	-	-	57.31	77.15	50.87	-	61.2	-	57.18
Chlamydiae sp RIFCSPHIGHO2_02_FULL_45_9	-	-	100.0	-	100.0	100.0	100.0	-	-	100.0	100.0
Chlamydiae sp RIFCSPLOWO2_02_FULL_45_22	-	-	100.0	100.0	100.0	100.0	100.0	-	100.0	100.0	100.0
Chlamydiae sp RIFCSPHIGHO2_12_FULL_44_59	-	-	100.0	100.0	100.0	100.0	100.0	-	100.0	100.0	100.0
Chlamydiae sp RIFCSPHIGHO2_01_FULL_44_39	-	-	100.0	100.0	100.0	100.0	100.0	-	100.0	100.0	100.0
Chlamydiae sp RIFCSPLOWO2_12_FULL_45_20	-	-	100.0	100.0	100.0	100.0	100.0	-	100.0	100.0	100.0
Chlamydiae sp RIFCSPLOWO2_01_FULL_44_52	-	-	100.0	100.0	100.0	100.0	100.0	-	100.0	100.0	100.0
Simkania negevensis Z	-	-	44.83	49.66	39.9	70.2	41.16	-	47.82	48.39	53.49
Rhabdochlamydia helveticae T3358	-	-	45.19	47.56	37.98	70.43	-	-	49.26	44.04	54.44
Chlamydiae sp Ga0074140	-	-	47.96	53.64	41.01	71.91	40.66	-	51.58	-	54.12
Chlamydiae sp GWA2_50_15	-	-	47.39	-	42.09	70.85	-	-	48.15	-	-
Chlamydiae sp RIFCSPHIGHO2_02_FULL_49_29	-	-	47.56	-	42.09	71.19	36.75	-	48.15	-	-
Chlamydiae sp RIFCSPLOWO2_02_FULL_49_12	-	-	47.39	-	42.09	-	36.75	-	48.32	-	-
Chlamydiae sp RIFCSPHIGHO2_12_FULL_49_32	-	-	47.39	-	42.09	71.19	36.75	-	48.15	-	-
Chlamydiae sp GWC2_50_10	-	-	47.39	-	42.09	-	-	-	48.15	-	-
Chlamydiae sp GWF2_49_8	-	-	47.39	-	42.09	-	-	-	52.46	-	-
Chlamydiae sp RIFCSPLOWO2_12_FULL_49_12	-	-	47.79	-	42.09	70.85	36.75	-	48.15	-	-
Chlamydiales sp SCGC AG_110_M15	-	-	-	-	43.58	69.7	-	-	-	-	-
Chlamydiales sp SCGC AG_110_P3	-	-	-	-	-	-	-	-	51.34	-	-
Estrella lausannensis CRIB_30	-	-	49.51	50.52	40.78	65.12	37.24	-	49.5	45.83	54.78
Criblamydia sequanensis CRIB_18	-	-	47.04	49.32	41.75	65.33	35.47	-	48.98	45.62	54.35
Waddlia chondrophila WSU 86_1044	-	-	-	52.58	42.07	69.15	37.24	-	50.42	46.95	50.35
Parachlamydiaceae sp HS_T3	-	-	51.11	49.32	44.56	70.2	39.49	-	49.67	47.32	53.27
Protochlamydia phocaeensis	-	-	52.46	52.39	40.15	69.77	38.82	-	53.45	47.09	52.46
Protochlamydia naegleriophila Diamant	-	-	53.12	51.32	39.8	69.33	41.56	-	51.51	42.6	49.88
Protochlamydia naegleriophila KNic	-	-	53.12	51.32	39.8	69.33	41.31	-	51.51	42.6	49.88
Protochlamydia sp R18 S13	-	-	53.6	50.64	40.88	67.44	38.0	-	50.59	41.52	50.35
Protochlamydia sp W_9	-	-	54.09	50.99	41.22	67.44	38.0	-	50.59	42.34	50.58
Protochlamydia amoebophila UWE25	-	-	53.6	51.28	41.22	67.44	38.54	-	50.59	41.52	50.12
Protochlamydia amoebophila EI2	-	-	53.6	-	40.98	67.44	36.87	-	50.59	41.52	-
Parachlamydia acanthamoebae Halls coccus	-	-	48.51	52.16	39.42	70.2	38.32	-	-	43.18	52.33
Parachlamydia acanthamoebae Bn9	-	-	48.51	51.81	39.42	70.53	38.32	-	49.83	43.18	52.09
Parachlamydia acanthamoebae OEW1	-	-	48.51	-	39.42	70.53	38.32	-	49.83	43.18	52.09
Parachlamydia acanthamoebae UV_7	-	-	48.51	51.81	39.42	70.53	38.32	-	49.83	43.18	52.09
Rubidus massiliensis	-	-	45.97	51.21	42.03	69.66	-	-	50.5	47.69	48.24
Chlamydia sp 32_24	-	-	45.97	50.98	42.03	69.66	-	-	50.5	47.83	48.24
Chlamydiales sp 38_26	-	-	50.37	51.43	42.75	73.74	40.92	-	51.76	46.19	53.77
Neochlamydia sp TUME1	-	-	49.51	-	41.79	71.04	40.71	-	51.34	49.76	50.82
Neochlamydia sp EPS4	-	-	49.51	-	42.03	70.71	41.48	-	51.51	50.24	51.52
Neochlamydia sp S13	-	-	49.75	-	42.75	70.71	40.97	-	51.17	50.71	51.75

Figure S7: Percentage of nucleotide and amino acid identity of the nine taxonomic markers as compared to Group 3 ('*Ca*. Enkichlamydiaceae'). The conservation of RpoN, PepF, Adk and HemL indicates that all six assemblies of group 3 ('*Ca*. Enkichlamydiaceae') are part of the same species. The conservation of protein 325 and FabI provide contradictory results regarding the classification of *Chlamydiae* sp. RIFCSPHIGHO2_12_FULL_49_9 as part of the same genus or not.

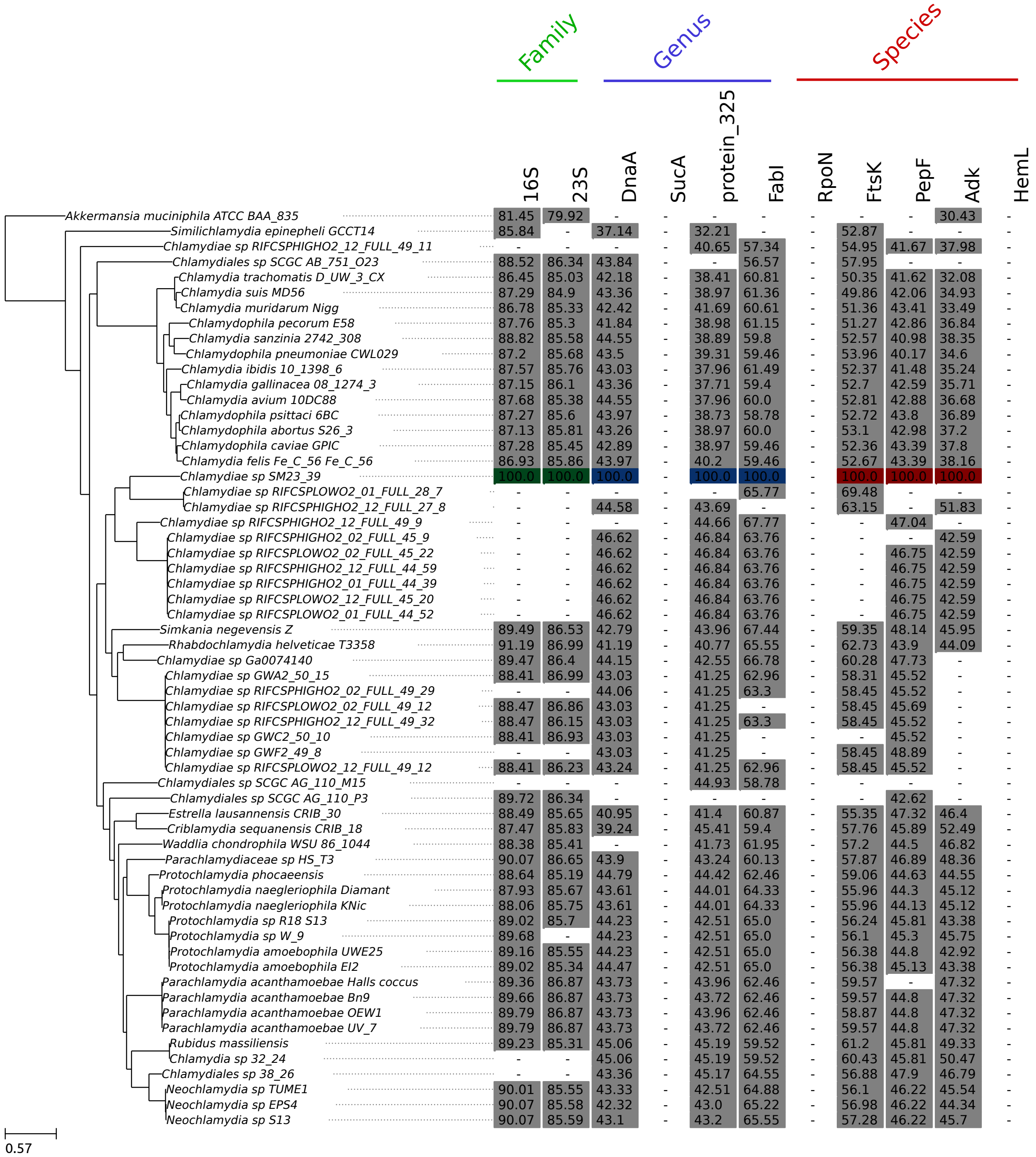

Figure S8: Percentage of nucleotide and amino acid identity of the nine taxonomic markers as compared to *Chlamydiae* bacterium SM23_39. The conservation of DnaA, protein 325 and FabI indicate that group 4 ('*Ca.* Limichlamydiaceae') can be subdivided in two genera.

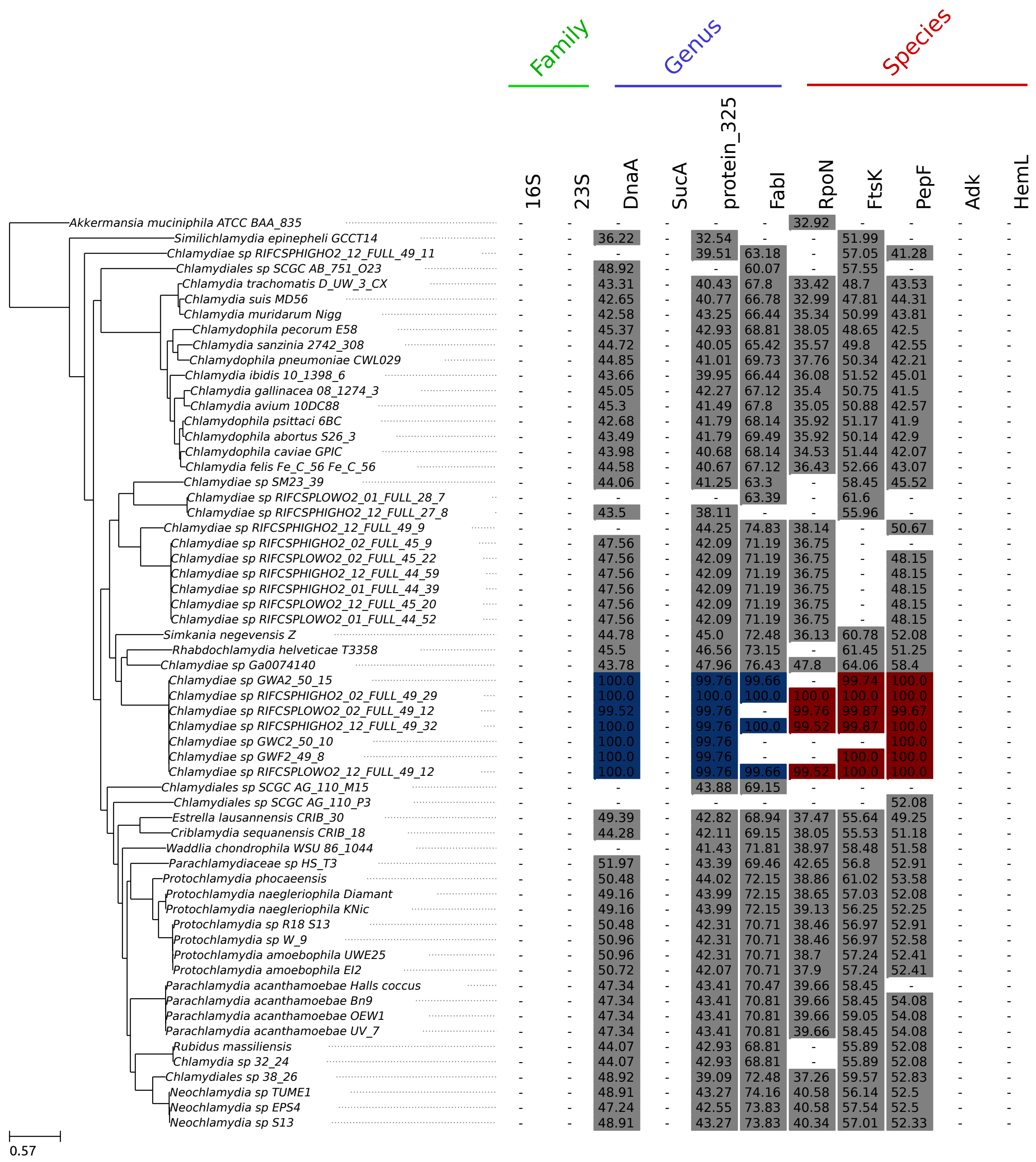

Figure S9: Percentage of nucleotide and amino acid identity of the nine taxonomic markers as compared to Group 5. The high level of conservation of RpoN, FtsK and PepF indicates that all 7 group 5 ('*Ca.* Arenachlamydiaceae') assemblies are part of the same species. The absence of 16S and 23S rRNA sequences precludes family-level classification based on the published scheme.

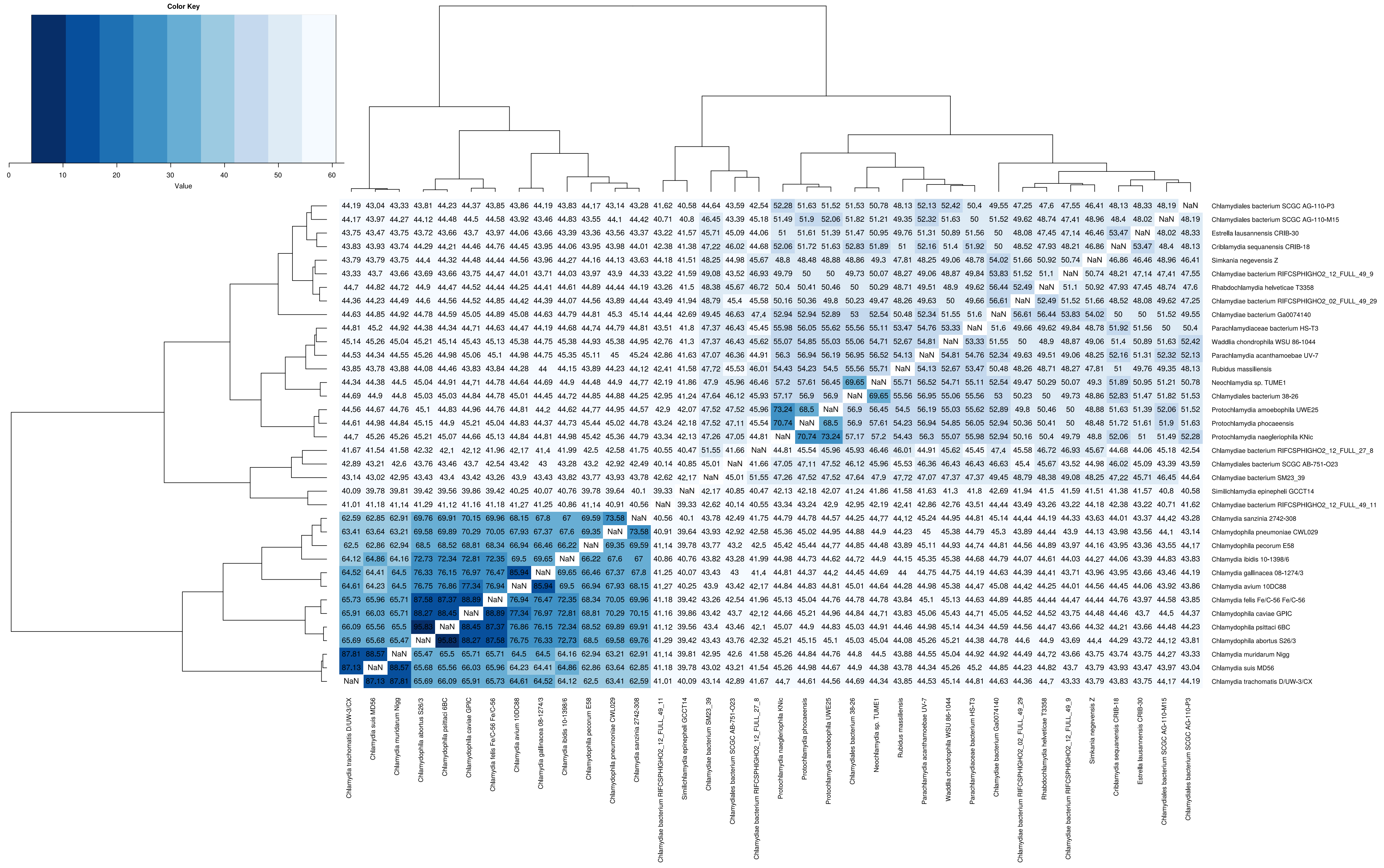

Figure S10: Median RBBH identity heat map. Median identity of reciprocal best blast hits calculated between one representative of each species and Candidatus species of the Phylum *Chlamydiae*

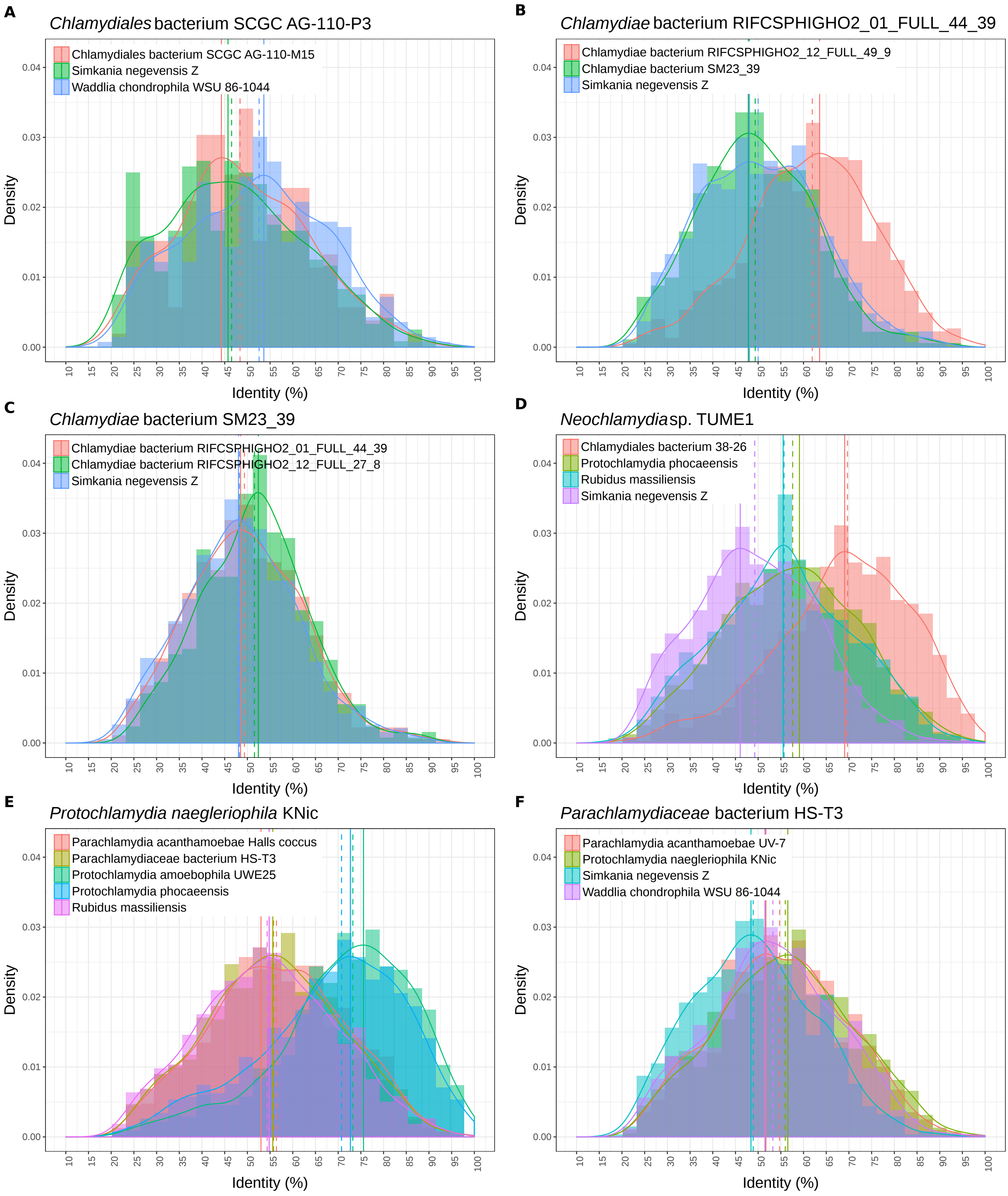
A
Chlamydiales bacterium SCGC AG-110-P3
Chlamydiales bacterium SCGC AG-110-M15
Simkania negevensis Z
Waddlia chondrophila WSU 86-1044
B
Chlamydiae bacterium RIFCSPHIGHO2_01_FULL_44_39
Chlamydiae bacterium RIFCSPHIGHO2_12_FULL_49_9
Chlamydiae bacterium SM23_39
Simkania negevensis Z
C
Chlamydiae bacterium SM23_39
Chlamydiae bacterium RIFCSPHIGHO2_01_FULL_44_39
Chlamydiae bacterium RIFCSPHIGHO2_12_FULL_27_8
Simkania negevensis Z
D
Neochlamydia sp. TUME1
Chlamydiales bacterium 38-26
Protochlamydia phocaeensis
Rubidus massiliensis
Simkania negevensis Z
E
Protochlamydia naegleriophila KNic
Parachlamydia acanthamoebae Halls coccus
Parachlamydiaceae bacterium HS-T3
Protochlamydia amoebophila UWE25
Protochlamydia phocaeensis
Rubidus massiliensis
F
Parachlamydiaceae bacterium HS-T3
Parachlamydia acanthamoebae UV-7
Protochlamydia naegleriophila KNic
Simkania negevensis Z
Waddlia chondrophila WSU 86-1044
Density
0.00
0.01
0.02
0.03
0.04
Identity (%)
10
15
20
25
30
35
40
45
50
55
60
65
70
75
80
85
90
95
100

Figure S11: RBBH identity distribution of newly identified clades. **A**) The two clades of marine *Chlamydiae* exhibit a median pairwise RBBH identity lower than 50%. Of note, those genomes are less than 50% complete. *S. negevensis* and *W. chondrohila* were added for comparison. **B**) Comparison of representatives of the two genera of group 3. *S. negevensis* and *Chlamydia* sp. SM_23-39 were added for comparison. **C**) The divergence of the two putative genus of group 4 is nearly as important as with other families **D**) *Chlamydia* sp. 28-36 most closely related to *Neochlamydia* sp. **E**) Comparison of the density distribution for different species of the *Protochlamydia* genus and comparison with representatives if two other genus. **F**) The *Parachlamydia* sp. HS-T3 shows high sequence divergence (median RBBH identity of about 55%) with representatives of other *Parachlamydiaceae* genus

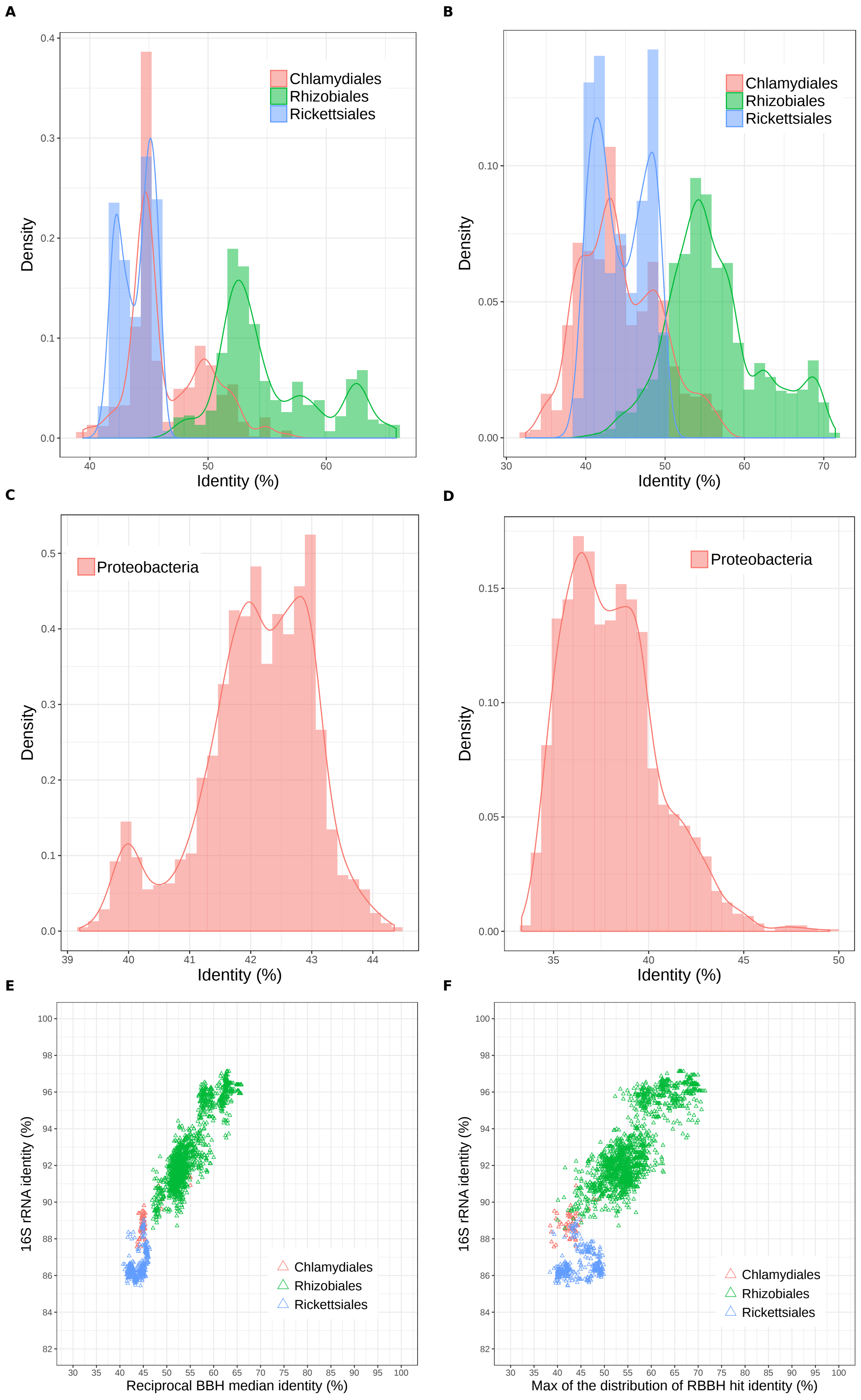
A
B
C
D
E
F
Density
Identity (%)
Chlamydiales
Rhizobiales
Rickettsiales
Proteobacteria
16S rRNA identity (%)
Reciprocal BBH median identity (%)
Max of the distribution of RBBH hit identity (%)

Figure S12: Density median RBBH and modal identity between families and orders, and complete chlamydia genomes comparisons. For this comparison, only a single order for the phylum *Chlamydiae* was considered. **A**) Distribution of the median identity between families within three different orders. **B**) Distribution of the median identity between families within three different orders. **C**) Distribution of the median identity between the *Rhizobiales* and *Rickettsiales* orders (alpha-proteobacteria). **D**) Distribution of the modal identity between the *Rhizobiales* and *Rickettsiales* orders (alpha-proteobacteria). **E**) The relationship between 16S rRNA sequence identity and the median RBBH identity between families within three distinct bacterial orders. Only complete genomes were included. **F**) The relationship between 16S rRNA sequence identity and the modal RBBH identity between families within three distinct bacterial orders. Only complete genomes were included

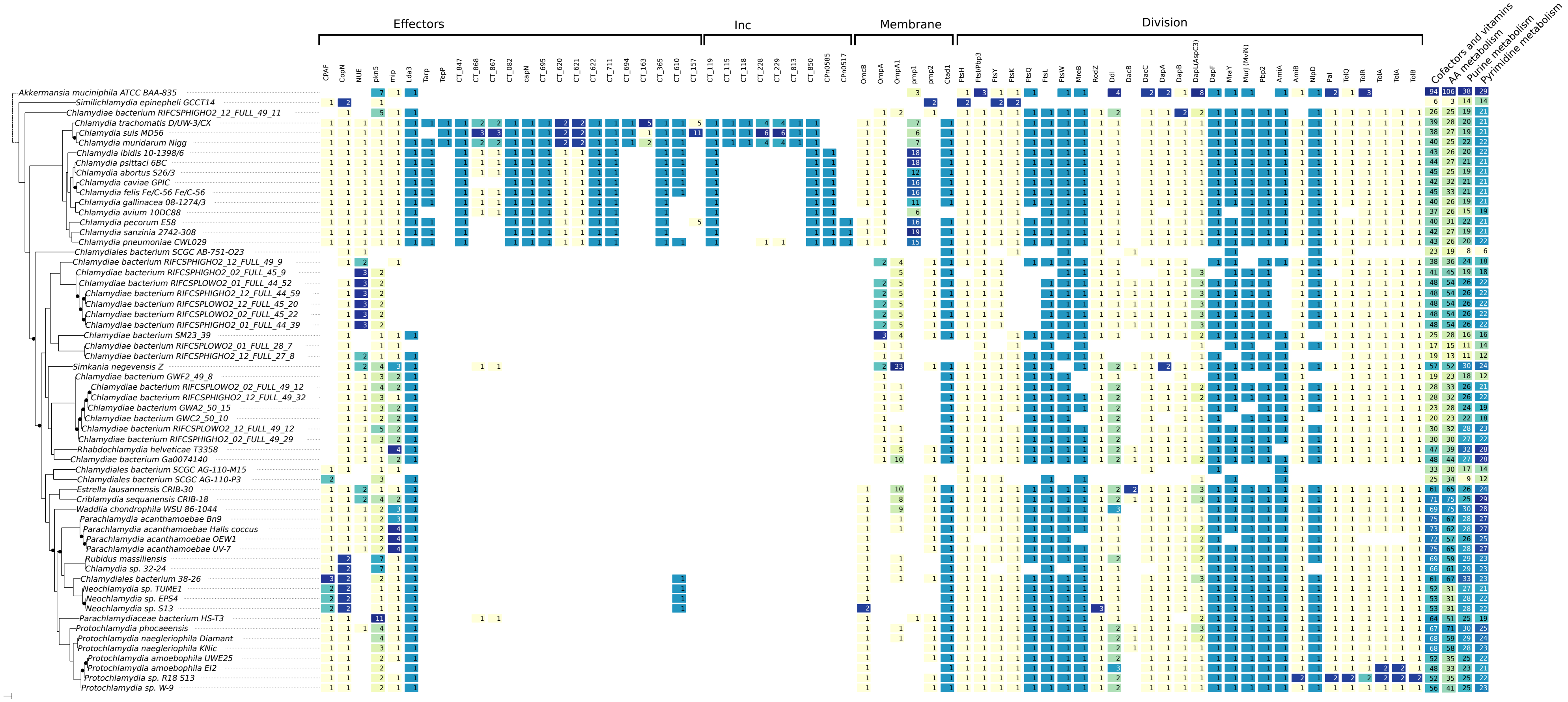

Figure S13: Identified homologs of effectors/inclusion membrane proteins, Membrane, and division proteins, as well as number of homologs identified in selected biosynthesis pathways. Data based on GhostKOALA annotation and clustering into orthologous groups with Orthofinder. Reference locus tags and KEGG accessions are indicated in table S1.

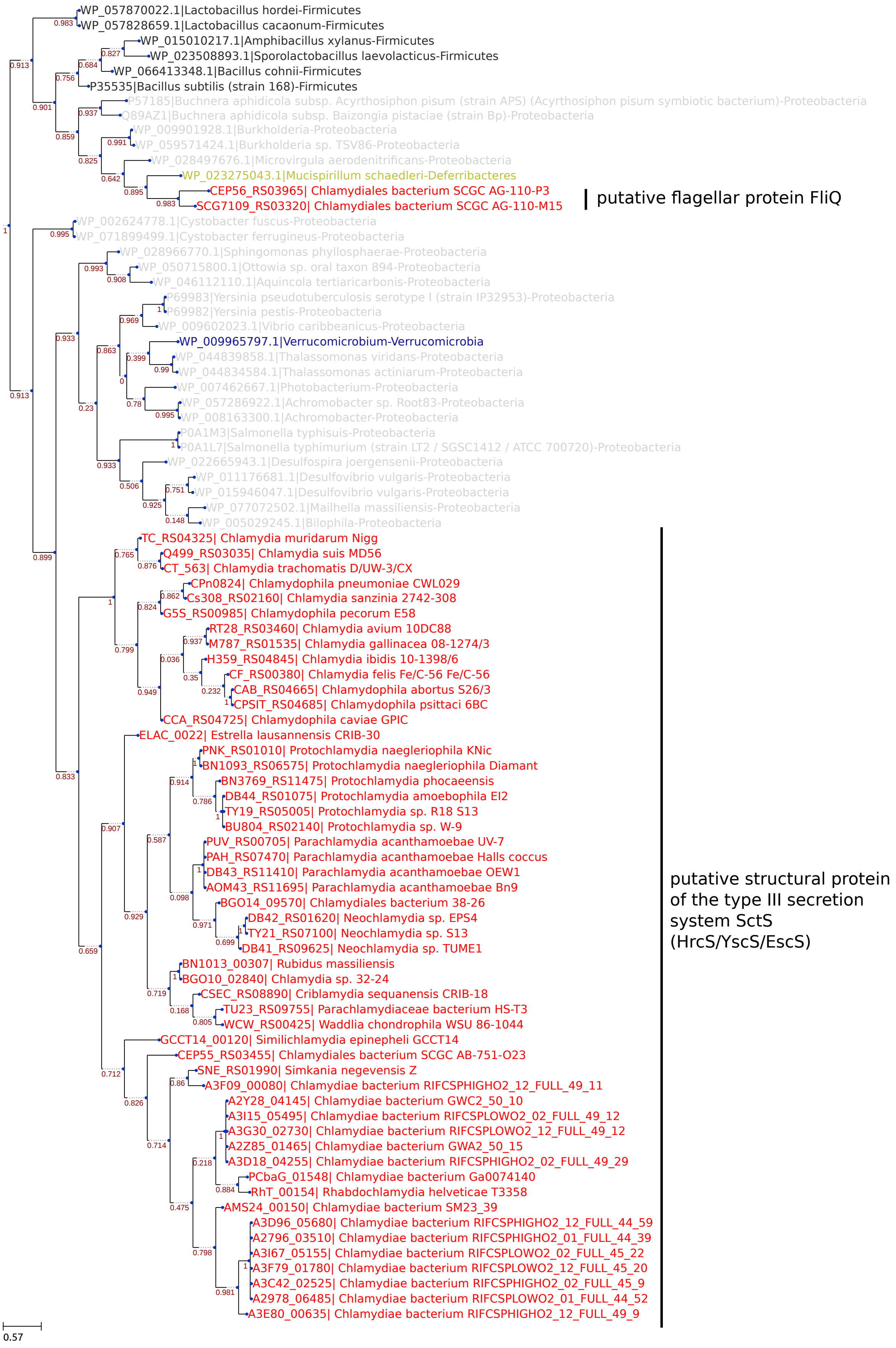

Figure S14: Phylogeny of the EscS/YscS/HrcS family type III secretion system export apparatus protein. The phylogeny includes the 2 closest RefSeq and SwissProt hits of each chlamydial sequence. Amino acid sequences were aligned with mafft version 7.058b and the phylogeny was reconstructed using Fasttree 2.1.9 with default parameters.

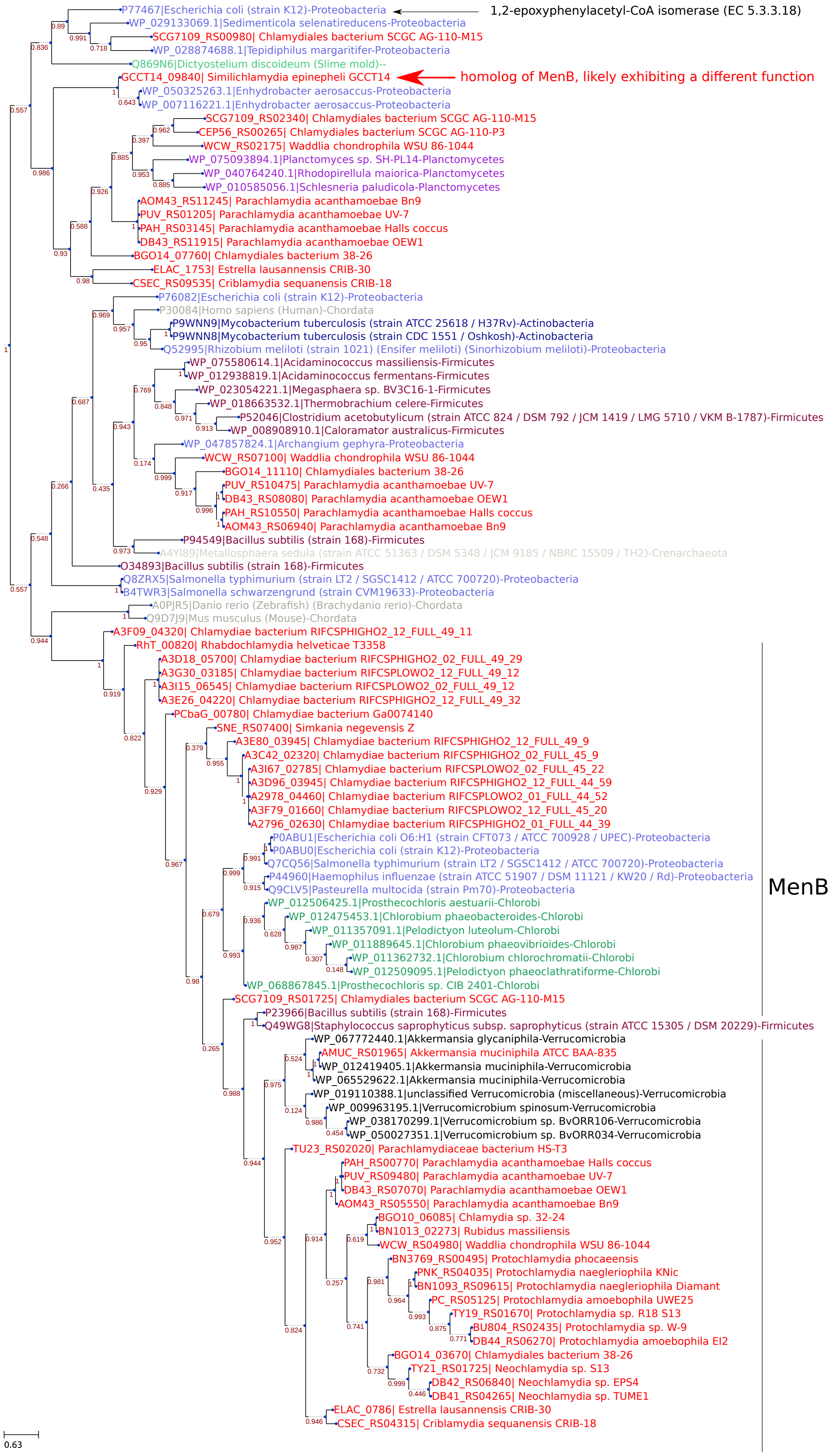

Figure S15: Phylogeny of the orthologous group including menB. The phylogeny includes the 2 best UniProt and 4 best RefSeq hits of each sequence of each protein of the orthologous groups. The menB homolog of *Chlamydiae* and their homologs in the genomes of *S. epinepheli* are not monophyletic. Amino acid sequences were aligned with mafft version 7.058b and the phylogeny was reconstructed using Fasttree 2.1.9 with default parameters.

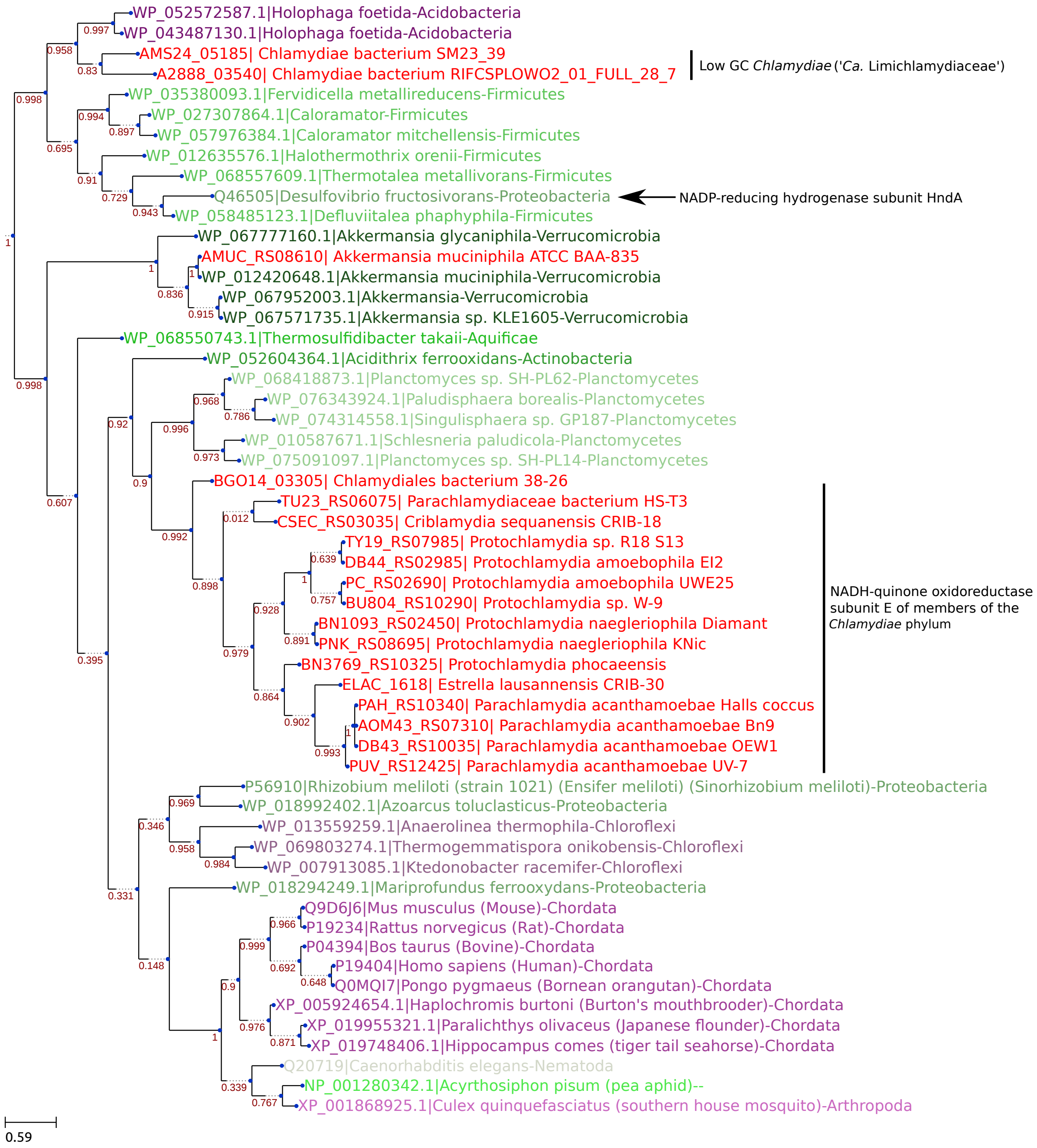

Figure S16: Phylogeny of the orthologous group including NADH-quinone oxidoreductase subunit E. The phylogeny includes the 2 best UniProt and 4 best RefSeq hits of each sequence of the orthologous groups. The NADH-quinone oxidoreductase subunit E orthologs from *Chlamydiae* and their homologs in low GC chlamydial genomes are not monophyletic. Amino acid sequences were aligned with mafft version 7.058b and the phylogeny was reconstructed using Fasttree 2.1.9 with default parameters.

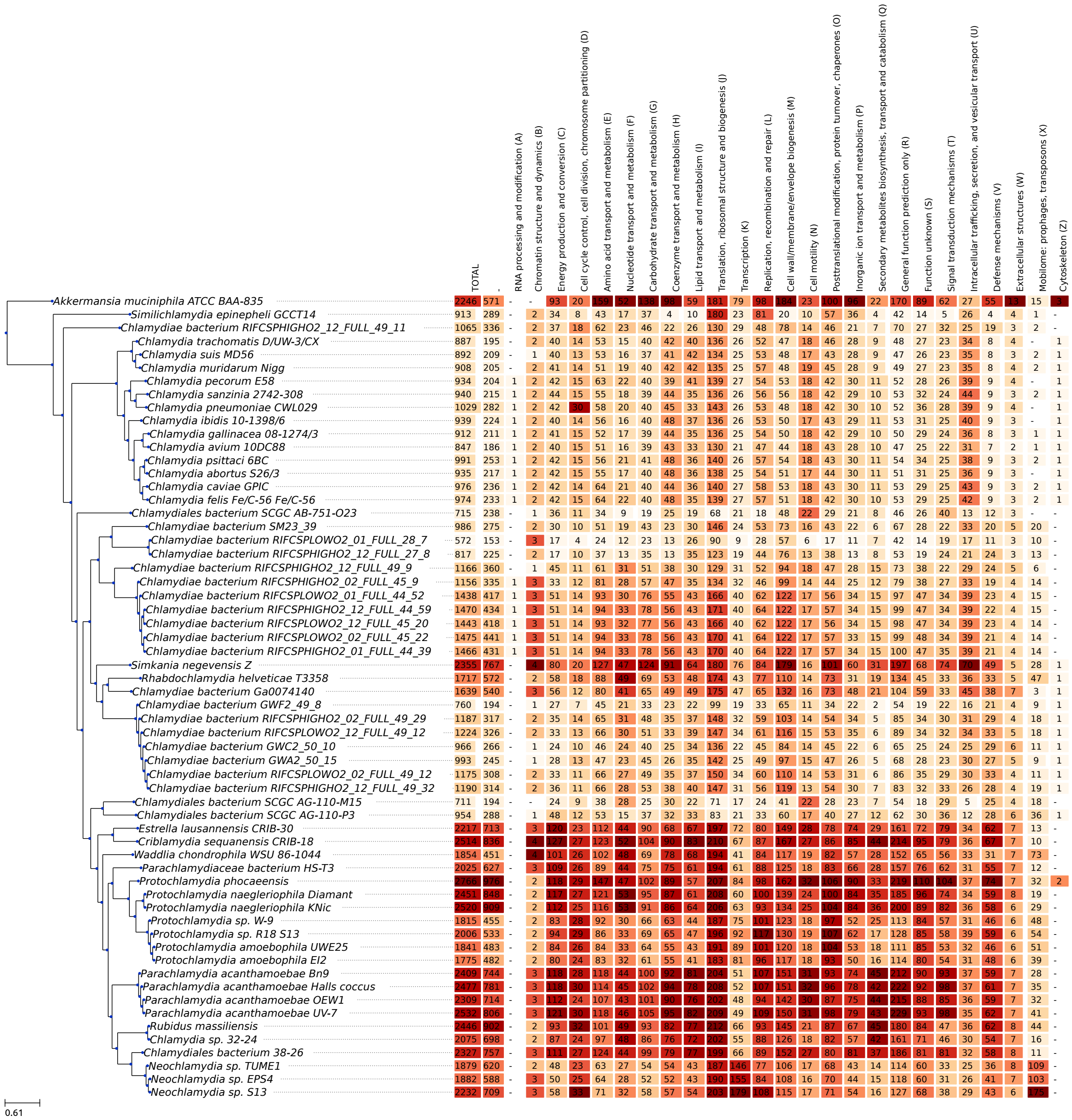

Figure S17: Classification of protein into COG categories. The red gradient reflect the number of annotated proteins with the corresponding COG category and was normalized for each colum (each COG category). The three *Neochlamydia* spp. encode a relatively large number of CDS (1879-2232), but a reduction in most COG categories except for mobile genetic elements (X) and transcription (K) as compared to closely related *Parachlamydiaceae*. The large increase in the transcription category in the 3 *Neochlamydia* genomes is due to the presence of a very large number of Leucine-rich repeat (LRR) proteins.

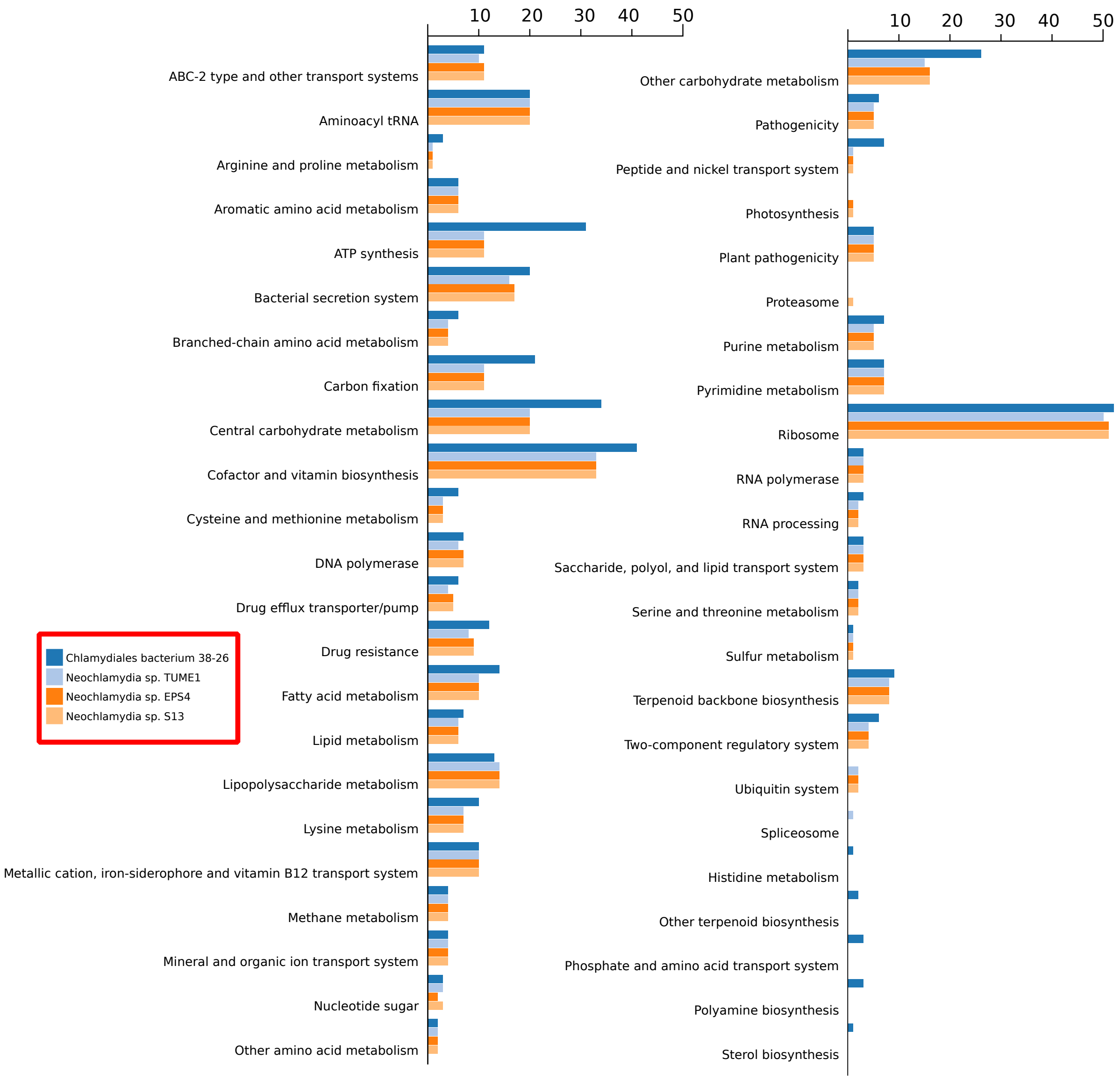

Figure S18: Comparison of KEGG functional categories for the Neochlamydia strains and their closest phylogenetic neigbour. Counts indicates the non-redundant number of Kegg Orthologs (KO) identified for each functional category. Gene losses are particularly important in categories such as ATP synthesis, amino acid metabolism and carbohydrate metabolism.
